# Synthesis and Characterisation of Multivariate Metal–Organic Frameworks for Controlled Doxorubicin Absorption and Release

**DOI:** 10.3390/molecules30091968

**Published:** 2025-04-29

**Authors:** Ahmed Ahmed, Andrey Bezrukov, Debobroto Sensharma, Ciaran O’Malley, Michael J. Zaworotko, Davide Tiana, Constantina Papatriantafyllopoulou

**Affiliations:** 1 SSPC Research Ireland Centre for Pharmaceuticals, Ireland; 2 School of Biological and Chemical Sciences, College of Science and Engineering, University of Galway, H91 TK33 Galway, Ireland; 3 Department of Chemical Sciences, Bernal Institute, University of Limerick, V94 T9PX Limerick, Ireland; 4 School of Chemistry, University College Cork, College Road, T12 K8AF Cork, Ireland

**Keywords:** metal–organic frameworks, drug delivery, functionalisation, multivariate MOFs, doxorubicin

## Abstract

The development of drug carriers with efficient absorption and controlled delivery properties is crucial for advancing medical treatments. Metal–organic frameworks (MOFs) with tunable porosity and a large surface area represent a promising class of materials for this application. Among them, **NUIG4** stands out as a biocompatible MOF that exhibits exceptionally high doxorubicin (Dox) absorption (1995 mg dox/g **NUIG4**) and pH-controlled release properties. In this study, we report the synthesis and characterisation of multivariate MOFs (**MV-NUIG4**), which are analogues of **NUIG4** that maintain the same topology while incorporating different functional groups within their framework. Eight new **MV-NUIG4** MOFs have been synthesised through in situ reactions of the corresponding 4-aminobenzoic acid derivative with 4-formylbenzoic acid. The compounds were thoroughly characterised using a range of techniques, including powder X-ray diffraction, infrared spectroscopy, ^1^H-NMR, and single-crystal X-ray crystallography. The experimental ratio of the reagents and ligand precursors for the synthesis of **MV-NUIG4** MOFs matched the ratio of the linkers in the final products. These structures incorporate additional functional groups, such as methyl and hydroxyl, in varying ratios. Computational modelling was used to provide further insight into the crystal structure of the MOFs, revealing a random distribution of the functional groups in the framework. The Dox absorption and release capacity of all analogues were studied, and the results revealed that all analogues displayed high drug absorption in the range of 1234–1995 mg Dox/g MOF. Furthermore, the absorption and release rates of the drug are modulated by the ratio of functional groups, providing a promising approach for controlling drug delivery properties in MOFs.

## 1. Introduction

Metal–organic frameworks (MOFs) [1,2,3,4,5,6,7,8] have attracted significant attention due to their unique structural features, as well as their potential to tackle challenges in the biomedical, environmental, and industrial fields [9,10,11,12,13,14,15,16,17]. The conceptual basis for MOFs traces back to the seminal work of Hoskins and Robson in 1989, who introduced the idea of infinite coordination polymers with permanent porosity, laying the foundation for this field [2]. Following this, the development of MOFs evolved into a vast field that has been shaped by many researchers globally. MOFs possess a large surface area and flexible and tunable structures, along with high porosity, which make them suitable for encapsulating a wide variety of guest molecules. As such, MOFs are promising candidates for applications in gas storage and separation [18,19,20,21,22,23], drug delivery [1,24,25,26,27,28], imaging [29,30], catalysis [31,32,33,34,35], sensing [36,37,38], and others]. Furthermore, they can protect large biomolecules from degradation and control their release, and they have advanced the fields of gene targeting, enzyme immobilization, and the crystallization and purification of proteins [39,40,41,42,43,44,45,46,47,48]. Recently, MOFs have also been explored as a potential solution to combat antibiotic resistance [24,25,49,50].

The intriguing properties and applications of MOFs have amplified the need to develop targeted synthesis approaches and deliberately integrate functional groups into their framework. These modifications aim to enhance MOF performance for specific uses, particularly in biomedical applications. In alignment with this, multivariate MOFs (MV-MOFs) [51,52,53,54,55,56,57] have gained significant attention due to their ability to introduce functionality to an MOF while maintaining the framework topology.

MV-MOFs are composed of various metal atoms and/or organic linkers, with the presence of these diverse components leading to a varied arrangement of functional groups while preserving the backbone structure [51,52,53,54,55,56,57]. Significantly, the properties of MV-MOFs are not simply a linear combination of the different functionalities, but rather it is the synergy between these functionalities that often leads to enhanced performance. One of the first examples of this is an MV-MOF5 analogue, which showcased a 400% increase in selectivity for CO_2_ over CO compared to analogues that consist of the link components separately [51,52].

Many families of MV-MOFs are now known, and most of them are being widely studied for their potential applications in gas absorption and separation, CO_2_ fixation, photocatalysis, and more [58,59,60,61,62,63,64]. Recently, they have also been explored in the field of drug delivery [63,64]. MV-MOFs are promising candidates in this context, as they enable fine-tuning of the drug release profile through the introduction of functional groups that regulate interactions between the framework and encapsulated drugs. A prime example of this is the family of MV-MIL101(Fe) MOFs [64], which are synthesised with three different linkers in varying ratios: benzenedicarboxylic acid (bdc), NH_2_-bdc, and 1,4-naphthalenedicarboxylic acid. Notably, increasing NH_2_ abundance resulted in slower Ibuprofen release but faster Dox and Rhodamine B release, with the control of the linker ratio enabling precise control of the release profile.

**Figure 1:**
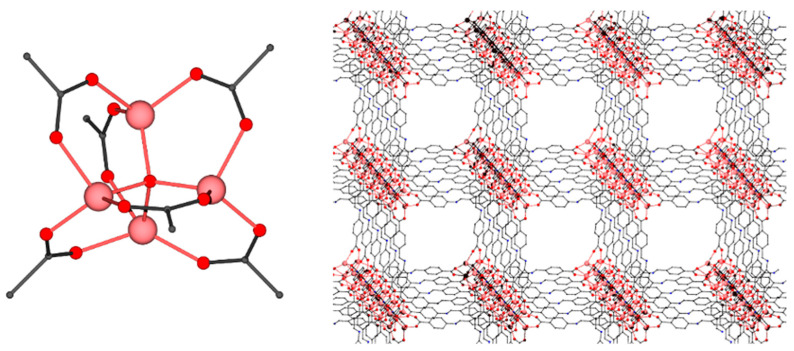
Crystal structure of **NUIG4**: (**left**) secondary building unit (SBU) highlighting the tetrahedral [Zn_4_O(COO)_6_] cluster, and (**right**) extended 3D framework. Visualised from published crystallographic data in ref. [1].

With the above in mind, we aimed to use the **NUIG4** MOF (Figure 1) as a basis to create MOFs with similar structural features but with different functional groups (**MV-NUIG4**). **NUIG4** has a Dox uptake capacity of 1995 mg Dox g^−1^, with a pH-dependent drug release rate that is greater under acidic conditions [1]. In particular, we aimed to synthesise **MV-NUIG4** MOFs with various functional groups and investigate their potential for controlled drug absorption and release. The functional groups were carefully selected to investigate various aspects of MOF–drug interactions, including hydrogen bonding capabilities, effects on pore accessibility, steric parameters, and aromatic interactions.

**Scheme 1:**
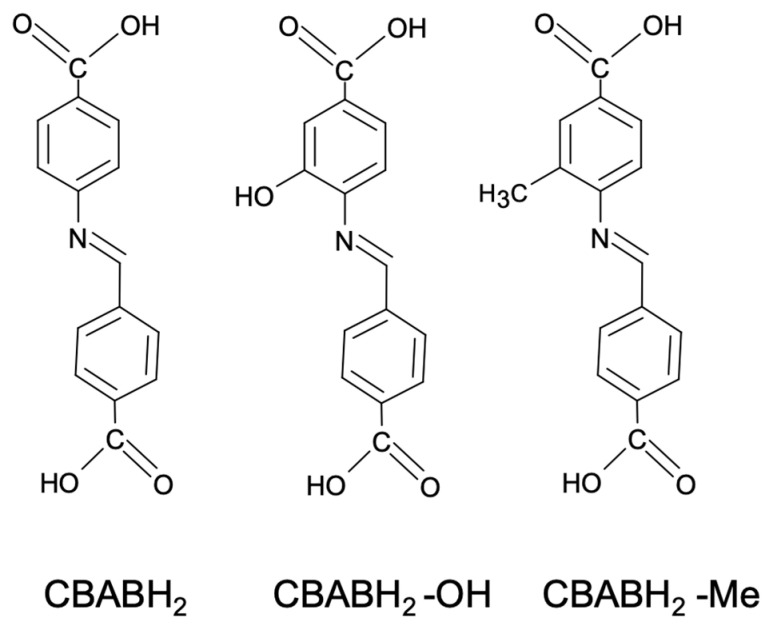
Schematic representation of the target linkers used in the synthesis of **MV-NUIG4** MOFs.

Herein, we report on the synthesis and characterisation of eight new members of the **MV-NUIG4** multivariate family of MOFs. The MOFs are isostructural to **NUIG4** and contain the ligands CBABH_2_ and CBABH_2_-OH, and CBABH_2_ and CBABH_2_-Me (Scheme 1) in varying ratios. A computational analysis confirmed a random distribution of the ligands throughout the frameworks. All **MV-NUIG4** analogues exhibited high Dox absorption, with their absorption and release profiles differing according to the type and proportion of functional groups within the framework, which provides a promising method for developing carriers with tailored drug delivery properties.

## 2. Results and Discussion

### 2.1. Synthetic Discussion

The possibility of isolating **MV-NUIG4** frameworks was investigated by reacting 4-aminobenzoic acid derivatives with 4-formylbenzoic acid and Zn(CH_3_CO_2_)_2_∙2H_2_O in DMF. Although the initial approach was to follow the **NUIG4** synthetic protocol, different reaction conditions and the use of modulators were explored.

Direct reactions between the linkers and Zn^2+^ sources produced amorphous materials or no solid products. By contrast, in situ reactions of the corresponding 4-aminobenzoic acid derivative with 4-formylbenzoic acid and Zn(CH_3_CO_2_)_2_∙2H_2_O resulted in crystalline materials.

A series of functional groups were investigated (CH_3_, OH, OCH_3_, naphthyl, NO_2_, pyridyl, and F) to create both individual frameworks and MV-MOFs. These groups were selected to study different MOF–drug interactions, including hydrogen bonding (hydroxy, fluoro, nitro, and pyridyl), pore accessibility (methyl), aromatic interactions (naphthyl), and steric effects (methoxy). Only CBABH_2_, CBABH_2_-OH, and CBABH_2_-Me successfully formed MV-MOFs, while bulkier linkers failed due to steric hindrance in the four-fold interpenetrated structure.

**Figure 2:**
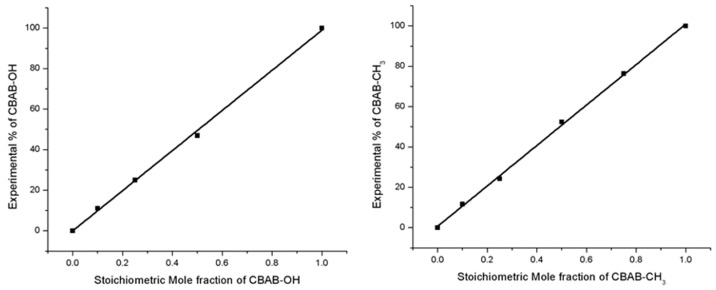
Plot of experimental vs. stoichiometric mole fraction of CBAB-OH (**left**) and CBAB-Me (**right**) in product. The line represents the best fit.

The experimental and product stoichiometry for the synthesis of the **MV-NUIG4** MOFs showed excellent linear correlation for CBAB-OH and CBAB-Me (regression factors: 0.9986 and 0.9988, respectively; Figure 2), demonstrating precise control over functional group ratios. Multiple sample analyses, including individual single crystal examinations, confirmed the formation of pure compounds rather than physical mixtures, ruling out linker bias.

### 2.2. Description of Structures

The structures of the **MV-NUIG4** MOFs were characterised with a variety of techniques, including ^1^H-NMR, single-crystal X-ray diffraction, powder X-ray diffraction (PXRD), and computational modelling.

The presence of two different types of ligands in the structure of **MV-NUIG4** and the exact framework composition were initially investigated through ^1^H-NMR on activated and digested samples. Upon digestion, the CBAB-R linkers decompose into 4-formylbenzoic acid and 4-amino-3-R-benzoic acid. Linker stoichiometry was determined by integrating characteristic proton signals of 4-amino-3-R-benzoic acid derivatives. The relative integration of signals at 7.59 ppm (4-amino-3-hydroxybenzoic acid, doublet, 2H), 7.38 ppm (4-aminobenzoic acid, doublet 2H), and 2.36 ppm (4-amino-3-methylbenzoic acid, singlet, 3H) provided insights into the linker ratios within the **MV-NUIG4** MOFs (Figures S1–S11 in the SI).

**Figure 3:**
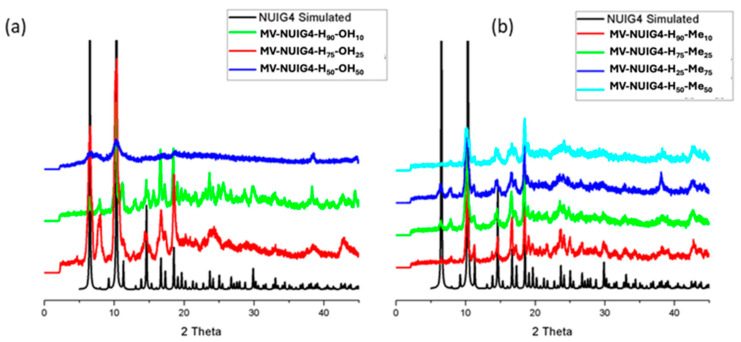
(**a**) PXRD patterns of **MV-NUIG4-H₉₀-OH₁₀**, **MV-NUIG4-H₇₅-OH₂₅**, and **MV-NUIG4-H₅₀-OH₅₀** compared to the simulated **NUIG4**; (**b**) PXRD patterns of **MV-NUIG4-H₉₀-Me₁₀**, **MV-NUIG4-H₇₅-Me₂₅**, **MV-NUIG4-H₂₅-Me₇₅**, and **MV-NUIG4-H₅₀-Me₅₀** compared to the simulated **NUIG4**.

Single-crystal X-ray diffraction analysis revealed that the **MV-NUIG4** MOFs are isostructural with **NUIG4**, as the unit cell parameters of **NUIG4** (P4_1_, a = b = 19.146, c = 19.117) were closely matched by the functionalised variants **MV-NUIG4-H_90_-OH_10_** (P4_1_, a = b = 19.15, c = 19.14) and **MV-NUIG4-H_75_-OH_25_** (P4_1_, a = b = 19.16, c = 19.12). PXRD analysis was employed to further confirm the crystallinity of the **MV-NUIG4-H_x_-OH_y_-Mez** framework and its structural similarity to **NUIG4** (Figure 3). The experimental PXRD patterns of the **MV-NUIG4** MOFs were compared with that of **NUIG4**, confirming structural similarity. Notably, framework crystallinity showed an inverse relationship with increasing values of y and z, which was attributed to enhanced structural disorder resulting from the introduction of OH and Me groups [51,52].

**Figure 4:**
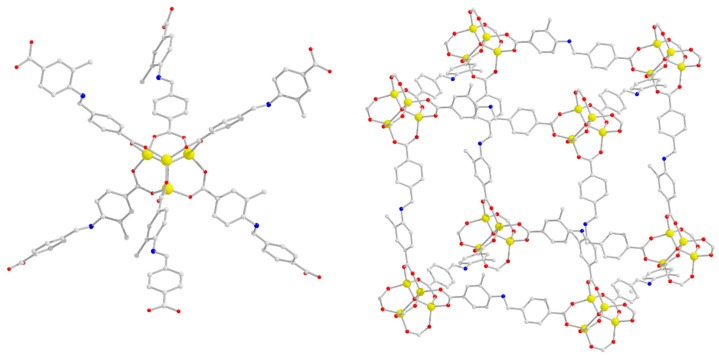
Representations of the tetrahedral repeating unit (**left**) and the 3D structure (**right**) of **NUIG4-Me**. Colour code: N, dark blue; Zn, yellow; O, red; C, grey.

**Figure 5:**
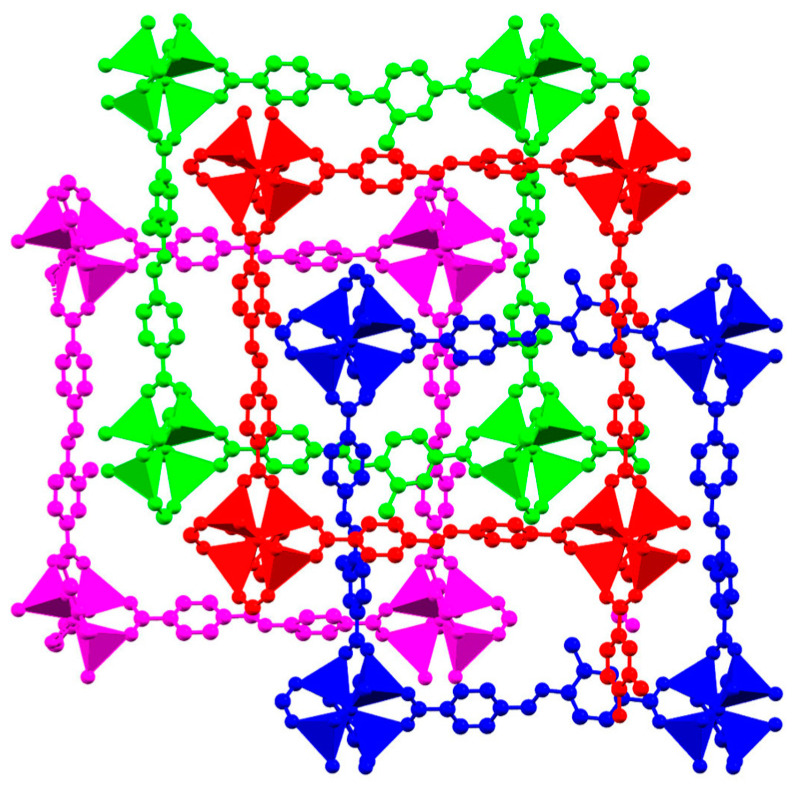
The topology of the four interpenetrating networks in **NUIG4-Me**.

Computational modelling was employed to further understand the crystal structure of **MV-NUIG4** MOFs as well as the ligand distribution within the framework. **NUIG4-Me** (Figure 4) crystallises in the tetragonal space group P4_1_, forming a cubic three-dimensional network constructed from tetrahedral [Zn_4_O(COO)_6_] secondary building units (SBUs). Like **NUIG4**, the structure exhibits four-fold interpenetration with interwoven networks (Figure 5). The solvent-accessible volume of **NUIG4-Me**, calculated using Mercury software, is 2843.6 Å^3^, which corresponds to 40.2% of the unit cell volume (7067.6 Å^3^). This value is lower than that of **NUIG4** (48.9%), which is expected due to the presence of additional functional groups in the framework and pores of **NUIG4-Me**.

Computational modelling provided insight into the distribution of the different functional groups within the framework of **MV-NUIG4**. In particular, simulations were performed on systems with varying OH content, specifically examining configurations with one and two OH groups out of twelve ligands (CIF files in the SI). The results revealed minimal energy differences among various OH distributions, with the maximum energy difference being 1.12 kcal/mol. This suggests a random distribution of OH groups, supporting the concept of a homogeneous solid solution. This finding aligns with several previous studies on mixed-linker MOF systems, which have demonstrated random distributions of functional groups within their framework [65,66,67]. The solvent-accessible volume of the **MV-NUIG4** MOFs bearing OH functional groups ranged from 43.6 to 44.0%, varying with the ratio of OH groups present in the framework. This value is larger than that of **NUIG4-Me** but smaller than that of **NUIG4**, as expected given the relative steric factors of the functional groups. The decrease in solvent-accessible volumes across **MV-NUIG4** analogues is consistent with increasing functionalisation, with values ranging from 44.0% to 40.2%, compared to 48.9% in **NUIG4**. This trend reflects steric and electronic effects of the substituents on pore geometry.

The introduction of methyl and hydroxyl substituents was observed to affect the crystallinity of the resulting frameworks, as evidenced by the broadening and intensity reduction of the PXRD peaks. This reduction in crystallinity likely reflects increased framework disorder or a decrease in long-range order, particularly in **NUIG4-OH**. In parallel, the calculated solvent-accessible volumes decreased with functionalisation. These effects, combined with potential hydrogen bonding or steric hindrance from the substituents, contribute to reduced pore accessibility.

### 2.3. Dox Absorption and Release Studies

The Dox uptake studies of the **MV-NUIG4** MOF family were conducted in MeOH:DMSO, 9:1, and the loadings of Dox were quantified using UV–vis spectroscopy (Figures S12–S19 in the SI). The quantified Dox loadings for **MV-NUIG4** match those observed for **NUIG4**, as shown in Table 1.

**Figure 6:**
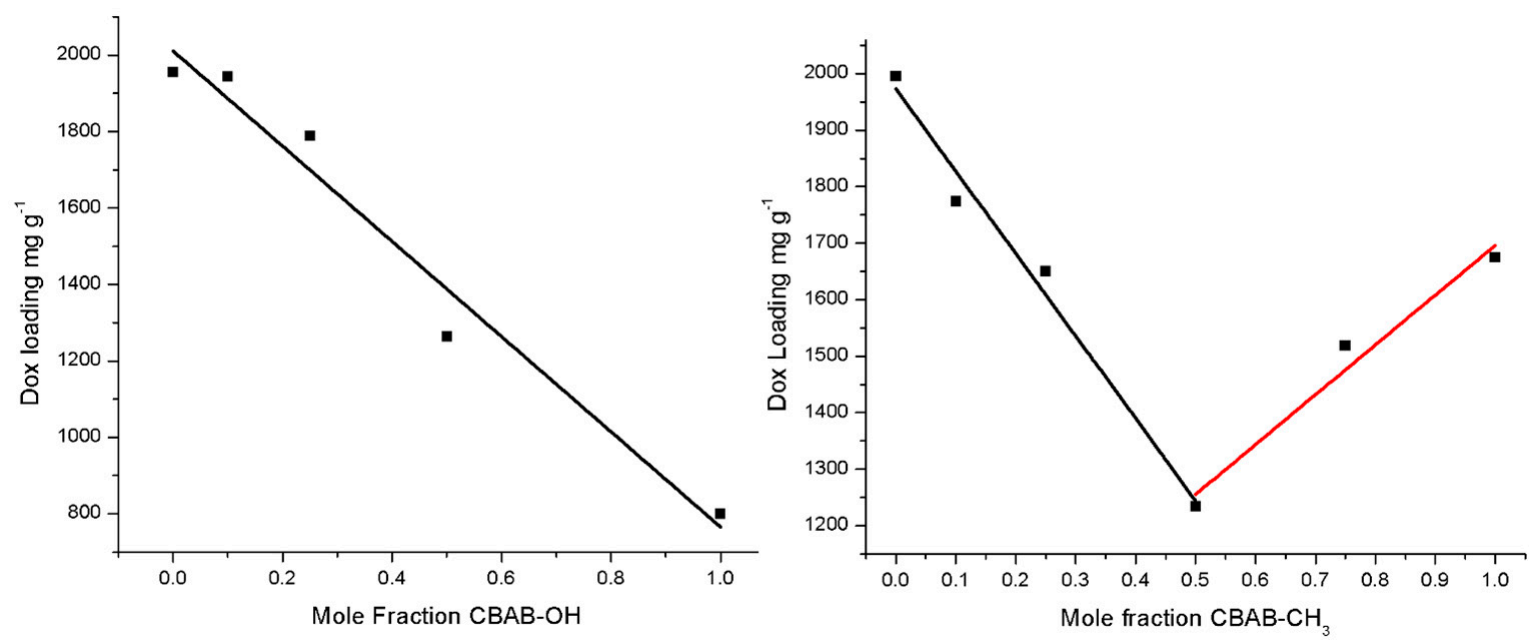
Plots showing the correlation between Dox loading and functional group composition in **MV-NUIG4** frameworks (**MV-NUIG4-H_x_-OH_y_**, (**left**); **MV-NUIG4-H_x_-Me_z_**, (**right**)).

The MOFs showed decreased Dox loadings as the mole fraction of CBAB-OH and CBAB-Me increased. For **MV-NUIG4-H_x_-OH_y_**, there is an almost linear relationship between Dox loading and the CBAB-OH mole fraction (Figure 6, left), with a linear regression factor of 0.9596. The relationship between the CBAB-Me mole fraction and the Dox loading capacity in the **MV-NUIG4-H_x_-Me_z_** family forms a V-shaped profile (Figure 6, right), with **MV-NUIG4-H_50_-Me_50_** showing the lowest loading of 1234 mg Dox g^−1^. Frameworks that have CBAB-Me mole fractions closer to the parent MOFs **NUIG4** and **NUIG4-Me** display greater loading capacities. The linear fitting of the decreasing and increasing loading capacity data yielded regression factors of 0.9748 and 0.9462, respectively. The linear relationship between Dox loading and functionalisation in both **MV-NUIG4** families indicates that if the degree of functionalisation is known, it is possible to estimate the loading of functionalised **MV-NUIG4** frameworks. 

**Figure 7:**
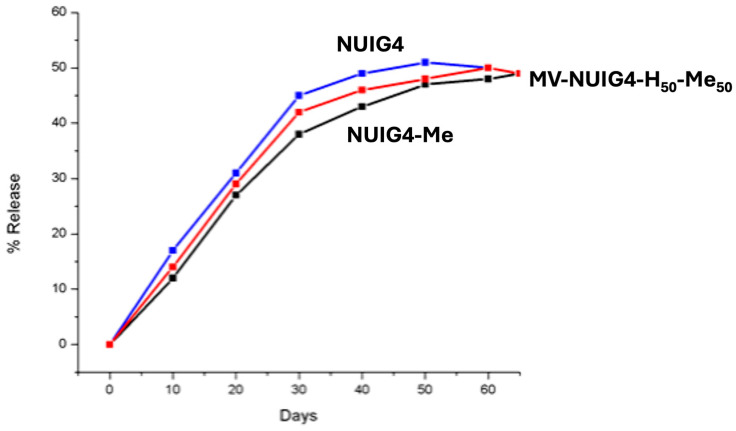
Dox release profiles of **MV-NUIG4** in acetate buffer (pH 5.5).

Drug release studies were conducted in both PBS solution (pH 7.4) and acetate buffer solution (pH 5.5), as shown in Figure 7. These frameworks exhibit a release behaviour akin to **NUIG4**, showcasing a slow release over a span of 10–20 days in water, and an accelerated release under acidic conditions. The quantity of Dox released in the **MV-NUIG4-H_x_-OH_y_** family decreases as the CBAB-OH mole fraction increases. This is related to the increased water stability of the MOFs due to hydroxylation *ortho* to aromatic imines. Indeed, hydroxylation *ortho* to aromatic imines has been utilized for enhancing water stability in both MOFs and COFs [68,69,70]. 1,2-Hydroxyl imines can undergo oxidative cyclisation, forming a benzoxazole group, which remains stable in water and across a wide pH spectrum. It was possible to modulate the release from 49% to 32% after 40 days by increasing the CBAB-OH mole fraction.

The **MV-NUIG4-H_x_-Me_z_** family exhibits a release profile similar to that of **NUIG4**, but with slower kinetics. As the CBAB-Me mole fraction increases, the initial release rate decreases. In particular, after 10 days, **NUIG4** releases 17% of the drug load, while **NUIG4-Me** releases 12%. This can be attributed to the reduced pore accessibility, as well as increased hydrophobicity due to the growing presence of methyl groups, which may reduce pore accessibility for H_2_O molecules. Despite the decreased initial release rate, the **MV-NUIG4-H_x_-Me_z_** frameworks eventually release the same quantity of Dox as **NUIG4** after 55 days. Therefore, the **MV-NUIG4-H_x_-Me_z_** MOF family offers a method to control the initial rate of Dox release by adjusting the proportion of Me groups in the framework.

To gain deeper insight into the release mechanism, the experimental data were analysed using the Higuchi and Korsmeyer–Peppas kinetic models (Figure S28) [71,72,73]. Both **NUIG4-Me** and **MV-NUIG4-H_50_-Me_50_** exhibited a strong linear correlation in the Higuchi model (R^2^ = 0.99 and 0.95, respectively), indicating that diffusion is a dominant factor governing the drug release process. However, fitting the data to the Korsmeyer–Peppas model revealed release exponents (*n*) of 0.79 and 0.74 for **NUIG4-Me** and **MV-NUIG4-H_50_-Me_50_**, respectively, which fall within the range of anomalous (non-Fickian) transport. This suggests that, in addition to diffusion, other processes such as framework relaxation or partial structural breakdown may contribute to the observed drug release. Such behaviour is common in porous coordination materials and highlights the intricate interplay between framework composition and drug release kinetics in MOFs.

## 3. Materials and Methods

### 3.1. Materials, and Physical and Spectroscopic Measurements

All chemicals used were commercially available and used without further purification. All procedures were conducted under aerobic conditions.

Elemental analyses (C, H, N) were performed by the in-house facilities of University of Galway. FTIR spectra (4000–400 cm^−1^) were recorded using a Perkin Elmer 16PC FT-IR spectrometer, with samples prepared as KBr pellets. PXRD data were collected using an Inex Equinox 6000 diffractometer at room temperature and pressure.

### 3.2. Compound Synthesis

#### 3.2.1. Synthesis of NUIG4-Me

4-Formylbenzoic acid (225 mg, 1.5 mmol) was dissolved in DMF (5 mL) within a glass scintillation vial. 4-Amino-3-methylbenzoic acid (226 mg, 1.5 mmol) was then added, resulting in a pale-yellow solution. Zn(CH_3_CO_2_)_2_·2H_2_O (330 mg, 1.5 mmol) was added, and the solution was sonicated for 2 min. The sealed vial was heated in an oven at 80 °C for 24 h, forming a white crystalline powder. The solvent was then decanted, and the powder was rinsed with DMF (2 × 5 mL). The yield was 41%, and the anal. calcd. (found) for **NUIG4-Me** was the following: C, 51.42 (51.87%); H, 2.97 (3.02%); N, 3.75 (3.28%).

#### 3.2.2. Synthesis of MV-NUIG4 Analogues

**MV-NUIG4** MOFs were synthesised similarly to **NUIG4** [1], but with varying mole fractions of 4-aminobenzoic acids. The general synthesis process described below was followed: In a 20 mL glass scintillation vial, 4-formylbenzoic acid (225 mg, 1.5 mmol) was dissolved in DMF (5 mL). To this, 4-aminobenzoic acid was added, followed by either 4-amino-3-hydroxybenzoic acid or 4-amino-3-methylbenzoic acid, with exact reagent quantities detailed in Table S1 of the SI. The solution was sonicated for 2 min. Zn(CH_3_CO_2_)_2_·2H_2_O (330 mg, 1.5 mmol) was added, and the solution was sonicated for 2 further minutes. The vial was sealed and placed in the oven at 80 °C for 24 h, yielding crystalline powders or small cubic crystals in the case of **MV-NUIG4-H_90_-OH_10_**, **MV-NUIG4-H_75_-OH_25_**, **MV-NUIG4-H_90_-Me_10_**, and **MV-NUIG4-H_75_-Me_25_**. The solvent was decanted, and the powders were washed with DMF (3 × 10 mL), while the crystals were kept in the mother liquor for single-crystal X-ray diffraction studies.

**MV-NUIG4-H_90_-OH_10_:** Yield: 27%. Anal. Calcd. (found): C, 49.86 (50.13%); H, 2.51 (2.60%); N, 3.88 (4.01%).

**MV-NUIG4-H_75_-OH_25_:** Yield: 20%. Anal. Calcd. (found): C, 49.53 (49.99%); H, 2.49 (2.52%); N, 3.85 (3.75%).

**MV-NUIG4-H_50_-OH_50_:** Yield: 15%. Anal. Calcd. (found): C, 48.99 (49.23%); H, 2.47 (2.58%); N, 3.81 (3.72%).

**MV-NUIG4-H_90_-Me_10_:** Yield: 32%. Anal. Calcd. (found): C, 50.22 (50.61%); H, 2.57 (2.68%); N, 3.88 (3.91%).

**MV-NUIG4-H_75_-Me_25_:** Yield: 35%. Anal. Calcd. (found): C, 50.42 (50.77%); H, 2.64 (2.79%); N, 3.86 (3.74%).

**MV-NUIG4-H_50_-Me_50_:** Yield: 30%. Anal. Calcd. (found): C, 50.76 (51.00%); H, 2.75 (2.93%); N, 3.82 (3.83%).

**MV-NUIG4-H_25_-Me_75_:** Yield: 35%. Anal. Calcd. (found): C, 51.09 (51.28%); H, 2.86 (2.94%); N, 3.78 (3.62%).

### 3.3. X-Ray Crystallography

Crystallographic data for **MV-NUIG4-H_90_-OH_10_**, **MV-NUIG4-H_75_-OH_25_**, **MV-NUIG4-H_90_-Me_10_**, and **MV-NUIG4-H_75_-Me_25_** were collected on an Oxford Diffraction Xcalibur CCD diffractometer using graphite-monochromatic Mo Kα radiation (*λ* = 0.71073 Å) at room temperature. Although the crystals diffracted poorly, reliable unit cell parameters were obtained for **MV-NUIG4-H_90_-OH_10_** (a = b = 19.15 Å, c = 19.14 Å, P4_1_) and **MV-NUIG4-H_75_-OH_25_** (a = b = 19.16 Å, c = 19.12 Å, P4_1_), confirming their structural similarity with **NUIG4** (a = b = 19.146 Å, c = 19.117 Å, P4_1_).

### 3.4. Crystal Structure Model Construction

Unit cell parameters of the frameworks were confirmed from powder X-ray diffraction pattern. Initial unit cell parameters for the frameworks were determined from positions of 10 observed peaks using indexing via DICVOL [74], which was implemented in DASH [75]. The unit cell parameters were refined for all frameworks via Pawley profile fit of the powder X-ray diffraction pattern using GSAS-II [76]. All calculations were performed using the open-source software Quantum Espresso v7.1 at the PBEsol level of theory [77]. Norm-conserving pseudopotentials were used, with an energy cutoff of 110 Ry. The unit cell parameters for the **MV-NUIG4** MOFs display significant similarities to **NUIG4**. A full geometry optimization was then performed on the primitive cell of the MOF, relaxing both the atomic positions and the lattice parameters. The standard convergence parameters were adopted.

### 3.5. NMR Studies

A total of 15 mg of activated MOF material was suspended in DMSO-d_6_ (500 μL). Then, 25 μL DCl (35 wt% in D_2_O) was added to the suspension. The suspension was sonicated for 5 min, yielding a clear solution. ^1^H-NMR spectra were recorded at 500 MHz using a Bruker spectrometer and were processed using Bruker Topspin software, with calibration against solvent signals in the literature.

### 3.6. MOF Activation and Drug Loading

**MV-NUIG4** MOFs were activated prior to drug uptake using the same procedure as for **NUIG4** [1]. A sample of MOF (20 mg) was stirred in DMF (15 mL) for 24 h, with DMF replacement after 4 and 8 h. The solid material was then collected by centrifugation at 4000 rpm for 5 min and stirred in MeOH (15 mL) for another 24 h, again replacing the MeOH after 4 and 8 h. Finally, the solid was collected by centrifugation and heated in an oven at 80 °C overnight.

Dox (0.04 g) was dissolved in MeOH:DMSO (9:1, 10 mL) and added to a centrifuge vial that contained **MV-NUIG4** (0.01 g). At specified time intervals, the solution was centrifuged, and 25 μL aliquots of the supernatant were removed and dissolved in MeOH (5 mL). The uptake of the drug was then monitored using UV–vis spectroscopy.

For the release studies, loaded **MV-NUIG4** (5 mg) was suspended in distilled H_2_O or PBS/5.5 pH sodium acetate buffer solution (10 mL) and stirred at 37 °C. At specified time intervals, the solution was centrifuged, and 100 mL aliquots of the solution were removed and diluted in distilled H_2_O or PBS solution (5 mL), and 100 mL of fresh solution was added to the vial. The release of Dox was then monitored using UV–vis spectroscopy.

## 4. Conclusions

The introduction of different functional groups to the **NUIG4** framework led to the synthesis and characterisation of eight new multivariate metal–organic frameworks (**MV-NUIG4**). The MOFs maintain the fundamental pcu topology of the parent **NUIG4** framework while incorporating different ratios of methyl and hydroxyl functional groups. **MV-NUIG4** MOFs were synthesised using an in situ approach, with reagent ratios accurately reflected in the final products. This methodology is particularly important because it enables the reliable production of frameworks with predictable structures and tunable functionalities.

The structural features of the **MV-NUIG4** MOFs were investigated using a variety of techniques, including ^1^H-NMR, single-crystal X-ray diffraction, powder X-ray diffraction, and computational modelling, which confirmed structural similarity to the parent MOF as well as a random distribution of the functional groups within the framework.

The **MV-NUIG4** analogues exhibited exceptional drug-loading capabilities, with doxorubicin absorption capacities ranging from 1234 to 1995 mg/g MOF, depending on the type and percentage of functional group present in the structure. This ability to modulate drug release rates through functional group ratios represents a particularly promising feature for pharmaceutical applications. Future studies will focus on investigating other functional groups and optimising their ratios to achieve targeted drug release profiles for Dox and other therapeutics.

## Figures and Tables

**Table 1:** Dox loading capacity of **MV-NUIG4** MOFs.

MV-NUIG4 Analogue	Dox Loading (mg g^−1^)
**NUIG4**	1995
**NUIG4-Me**	1675
**MV-NUIG4-H_90_-OH_10_**	1944
**MV-NUIG4-H_75_-OH_25_**	1788
**MV-NUIG4-H_50_-OH_50_**	1264
**MV-NUIG4-H_90_-Me_10_**	1773
**MV-NUIG4-H_75_-Me_25_**	1650
**MV-NUIG4-H_50_-Me_50_**	1234
**MV-NUIG4-H_25_-Me_75_**	1518

## Data Availability

The data presented in this study are available on request from the corresponding author.

## Supplementary Materials

The following supporting information can be downloaded at: https://www.mdpi.com/article/10.3390/molecules30091968/s1, Figure S1: ^1^H-NMR of digested **MV-NUIG4-H_75_-OH_25_**; Figure S2: ^1^H-NMR of digested **MV-NUIG4-H_50_-OH_50;_ Figure S3**: ^1^H-NMR of digested **MV-NUIG4-H_90_-OH_10;_** Figure S4: ^1^H-NMR of digested **MV-NUIG4-H_50_-Me_50;_** Figure S5: ^1^H-NMR of digested **MV-NUIG4-H_90_-Me_10_**; Figure S6: ^1^H-NMR of digested **MV-NUIG4-H_75_-Me_25_**; Figure S7: ^1^H-NMR of digested **MV-NUIG4-H_25_-Me_75_**; Figure S8: ^1^H-NMR of 4-aminobenzoic acid under digestion conditions; Figure S9: ^1^H-NMR of 4-amino-3-methylbenzoic acid under digestion conditions; Figure S10: ^1^H-NMR of 4-amino-3-hydroxybenzoic acid under digestion conditions; Figure S11: ^1^H-NMR of 4-formylbenzoic acid under digestion conditions; Figure S12: UV-Vis data for Dox uptake by **MV-NUIG4-H_50_-OH_50_***;* Figure S13: UV-Vis data for Dox uptake by **MV-NUIG4-H_90_-OH_10_**; Figure S14: UV-Vis data for Dox uptake by **MV-NUIG4-H_75_-OH_25_**; Figure S15: UV-Vis data for Dox uptake by **NUIG4-Me**; Figure S16: UV-Vis data for Dox uptake by **MV-NUIG4-H_50_-Me_50_**; Figure S17: UV-Vis data for Dox uptake by **MV-NUIG4-H_90_-Me_10_**; Figure S18: UV-Vis data for Dox uptake by **MV-NUIG4-H_75_-Me_25_**; Figure S19: UV-Vis data for Dox uptake by **MV-NUIG4-H_75_-Me_25_**; Figure S20: FTIR spectrum of **MV-NUIG4-H_50_-OH_50_**; Figure S21: FTIR spectrum of **MV-NUIG4-H_90_-OH_10_**; Figure S22: FTIR spectrum of **MV-NUIG4-H_90_-OH_10_**; Figure S23: FTIR spectrum of **MV-NUIG4-H_25_-OH_75_**; Figure S24: FTIR spectrum of **MV-NUIG4-H_50_-Me_50_**; Figure S25: FTIR spectrum of **MV-NUIG4-H_75_-Me_25_**; Figure S26: FTIR spectrum of **MV-NUIG4-H_90_-Me_10_**; Figure S27: FTIR spectrum of **NUIG4-Me**; Figure S28: Higuchi (left) and Korsmeyer–Peppas model fitting (right) of Doxorubicin release from **NUIG4-Me** and **MV-NUIG4-H_50_-Me_50_** (pH 5.5); Table S1: Quantities (mg) of 4-aminobenzoic acid and derivatives used in the synthesis of **MV-NUIG4** MOFs.
