# Supplementary material for: Synthesis and Characterisation of Multivariate Metal–Organic Frameworks for Controlled Doxorubicin Absorption and Release
Source: Molecules. 2025 Apr 29;30(9):1968. doi: 10.3390/molecules30091968 (PMC12073425; doi:10.3390/molecules30091968)
Supplement: Supplementary file 1 [file molecules-30-01968-s001.zip › molecules-3612799-supplementary material-send for convert/MV-NUIG4-SI.-R.docx]

SUPPORTING INFORMATION

**Synthesis and Characterisation of Multi-Variate Metal-Organic Frameworks for Controlled Doxorubicin Absorption and Release**

Ahmed Ahmed,^a,b^ Andrey Bezrukov,^c^ Debobroto Sensharma,^a,c^ Ciaran O’Malley,^b^ Michael J. Zaworotko,^a,c^ Davide Tiana, *^a,d^ and Constantina Papatriantafyllopoulou*^a,b^

1. SSPC Research Ireland Centre for Pharmaceuticals
2. School of Biological and Chemical Sciences, College of Science and Engineering, University of Galway, H91 TK 33 Galway, Ireland; E-mail address: [constantina.papatriantafyllopo@universityofgalway.ie](mailto:constantina.papatriantafyllopo@universityofgalway.ie); Tel: +353 91 493462.
3. Department of Chemical Sciences, Bernal Institute, University of Limerick, Limerick, V94T9PX, Republic of Ireland.
4. School of Chemistry, University College Cork, College Road, Cork, Ireland.

**Table S1**. Quantities (mg) of 4-aminobenzoic acid and derivatives used in the synthesis of **MV-NUIG4** MOFs.

| **MV-NUIG4** | **4-aminobenzoic acid** | **4-amino-3-hydrobenzoic acid** | **3-methylbenzoic acid** |
| --- | --- | --- | --- |
| **MV-NUIG4-H_90_-OH_10_** | 185 | 23 | *-* |
| **MV-NUIG4-H_75_-OH_25_** | 154 | 57 | - |
| **MV-NUIG4-H_50_-OH_50_** | 103 | 115 | - |
| **MV-NUIG4-H_90_-Me_10_** | 185 | - | 23 |
| **MV-NUIG4-H_75_-Me_25_** | 154 | - | 57 |
| **MV-NUIG4-H_50_-Me_50_** | 103 | - | 154 |
| **MV-NUIG4-H_25_-Me_75_** | 51 | - | 170 |


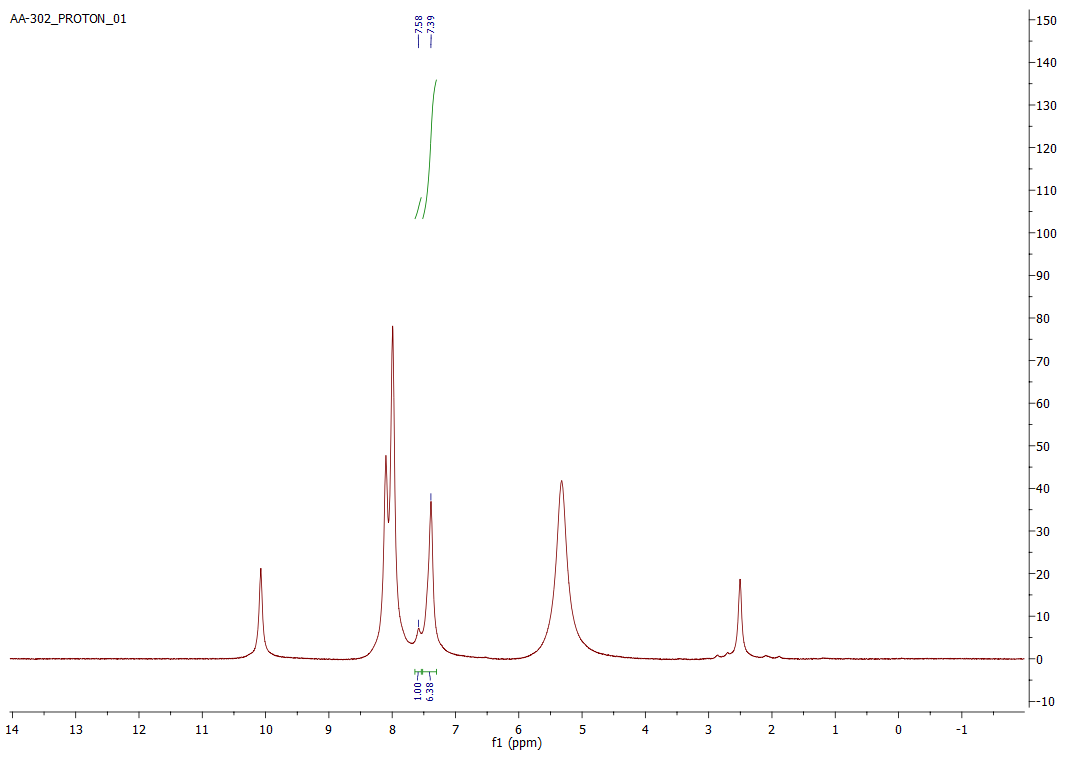


**Figure S1.** ^1^H-NMR of digested **MV-NUIG4-H_75_-OH_25_**.


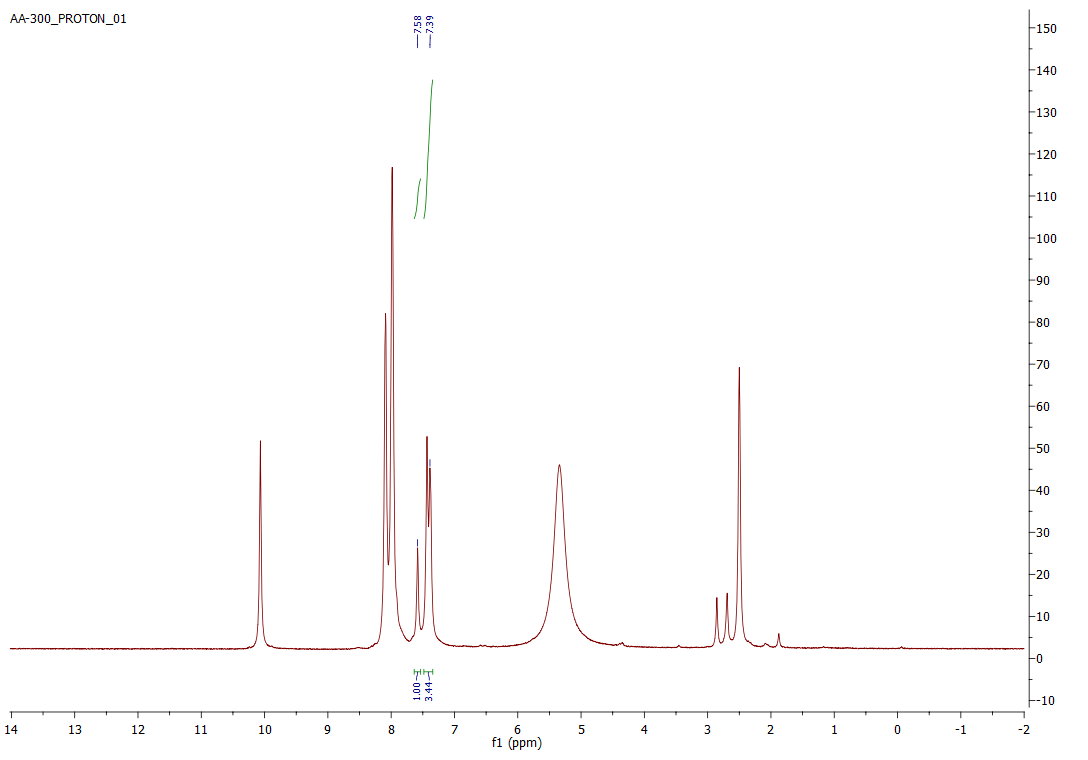


**Figure S2.** ^1^H-NMR of digested **MV-NUIG4-H_50_-OH_50_**.


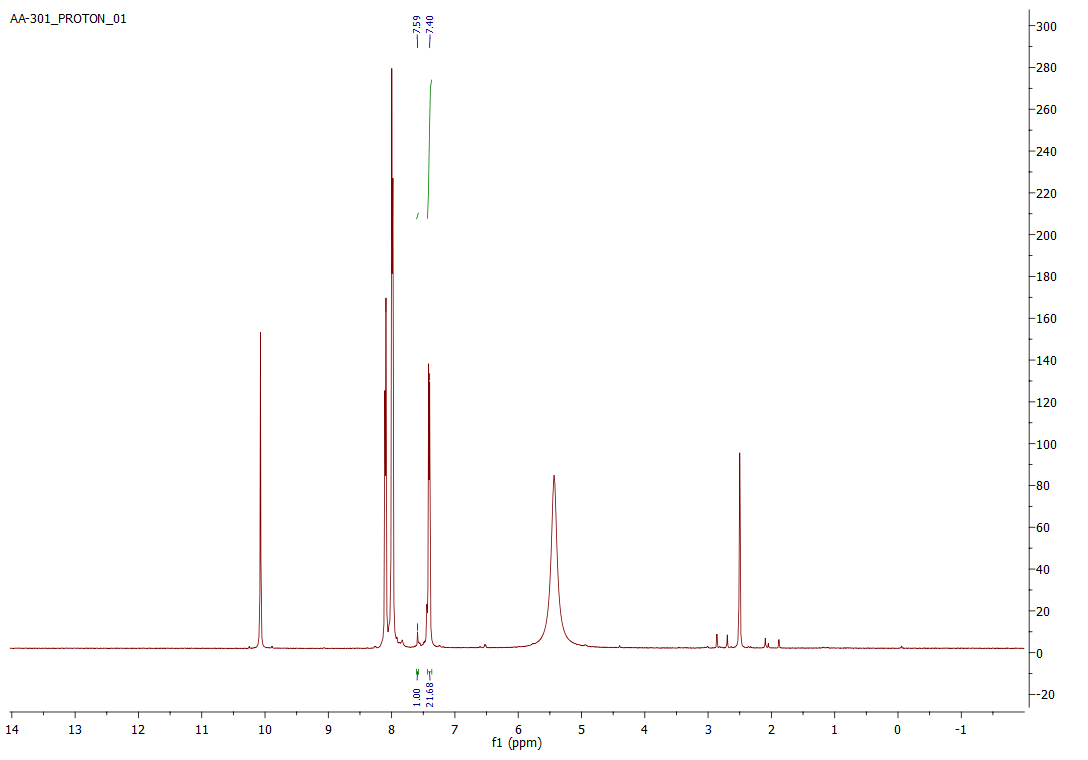


**Figure S3.** ^1^H-NMR of digested **MV-NUIG4-H_90_-OH_10_**.


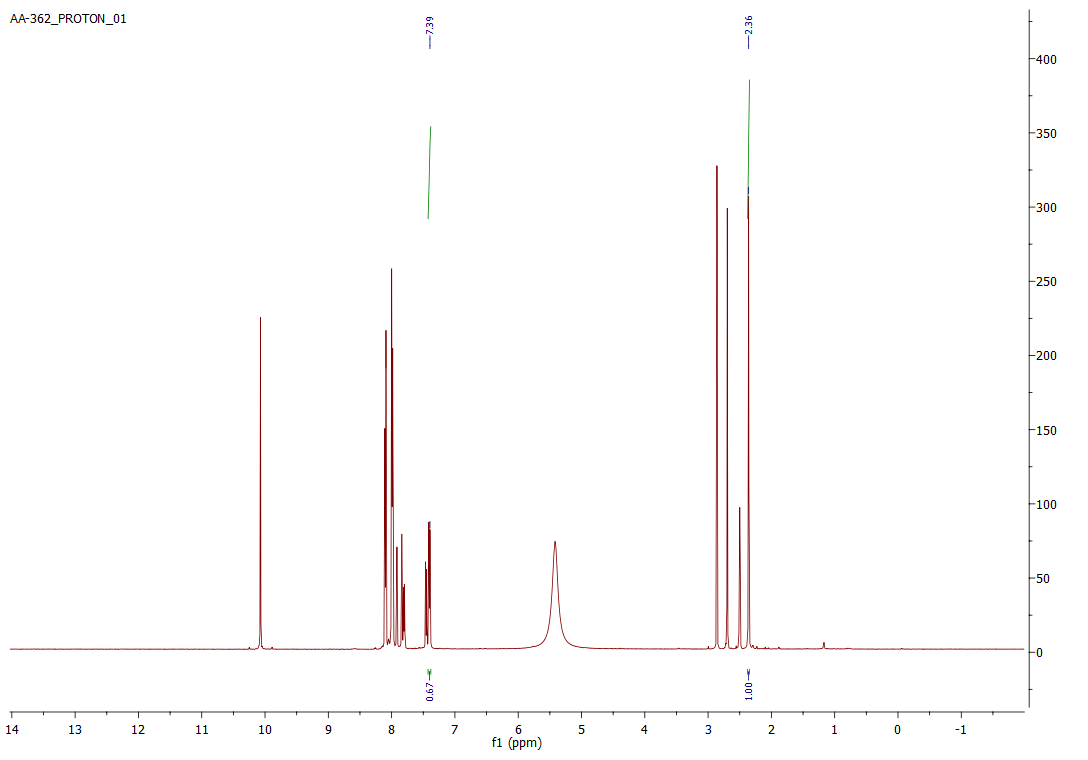


**Figure S4.** ^1^H-NMR of digested **MV-NUIG4-H_50_-Me_50_**.


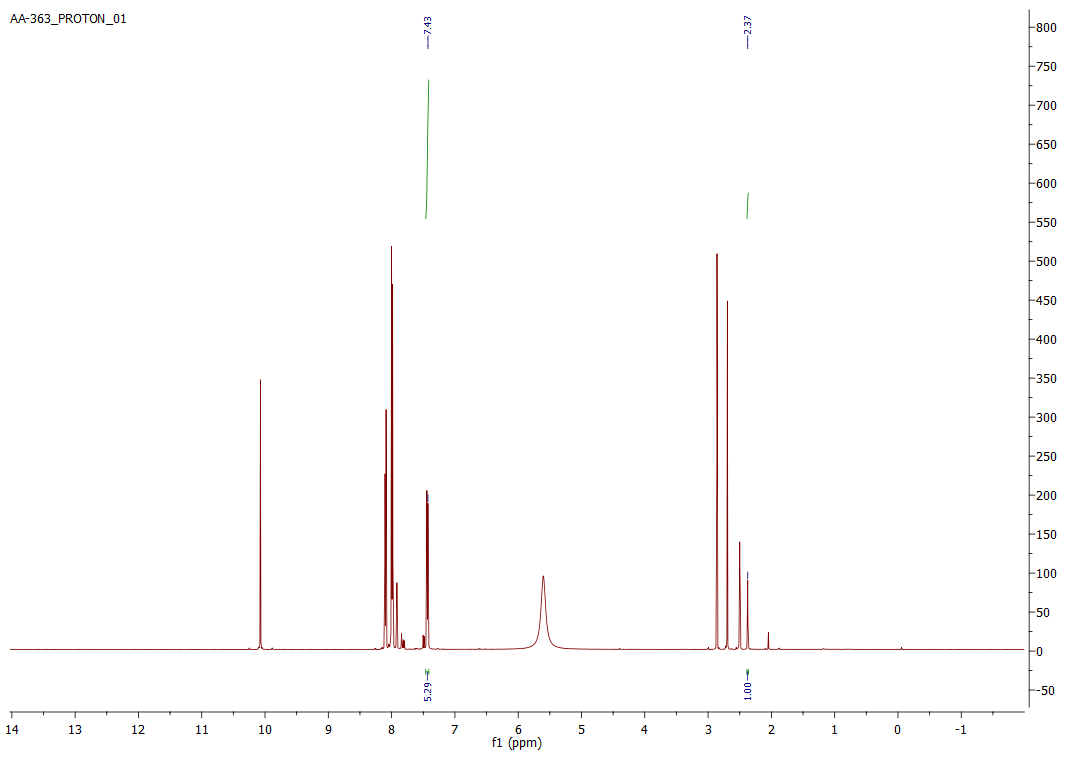


**Figure S5.** ^1^H-NMR of digested **MV-NUIG4-H_90_-Me_10_**.


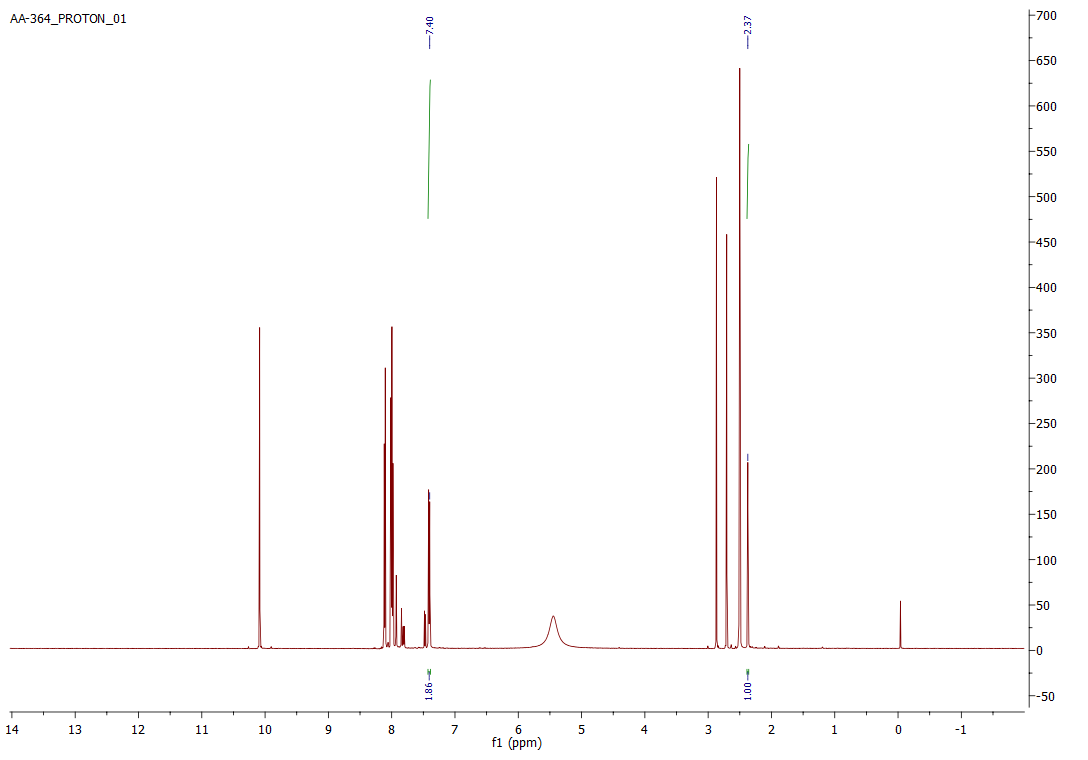


**Figure S6.** ^1^H-NMR of digested **MV-NUIG4-H_75_-Me_25_**.


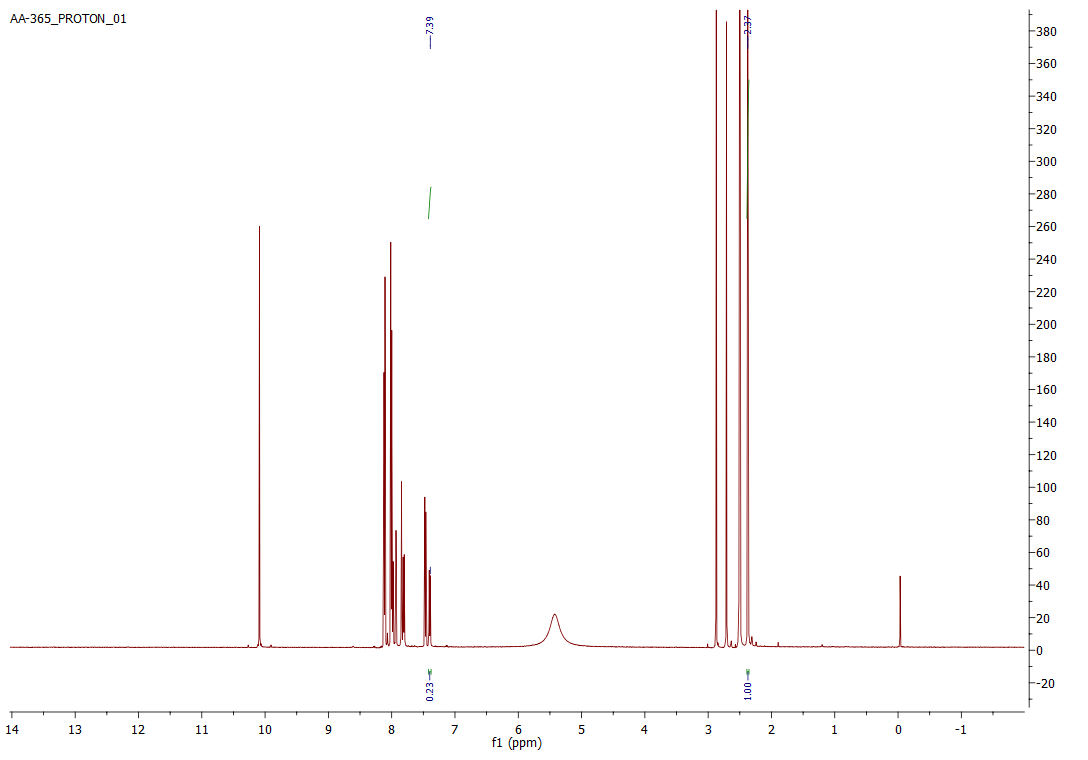


**Figure S7.** ^1^H-NMR of digested **MV-NUIG4-H_25_-Me_75_**.


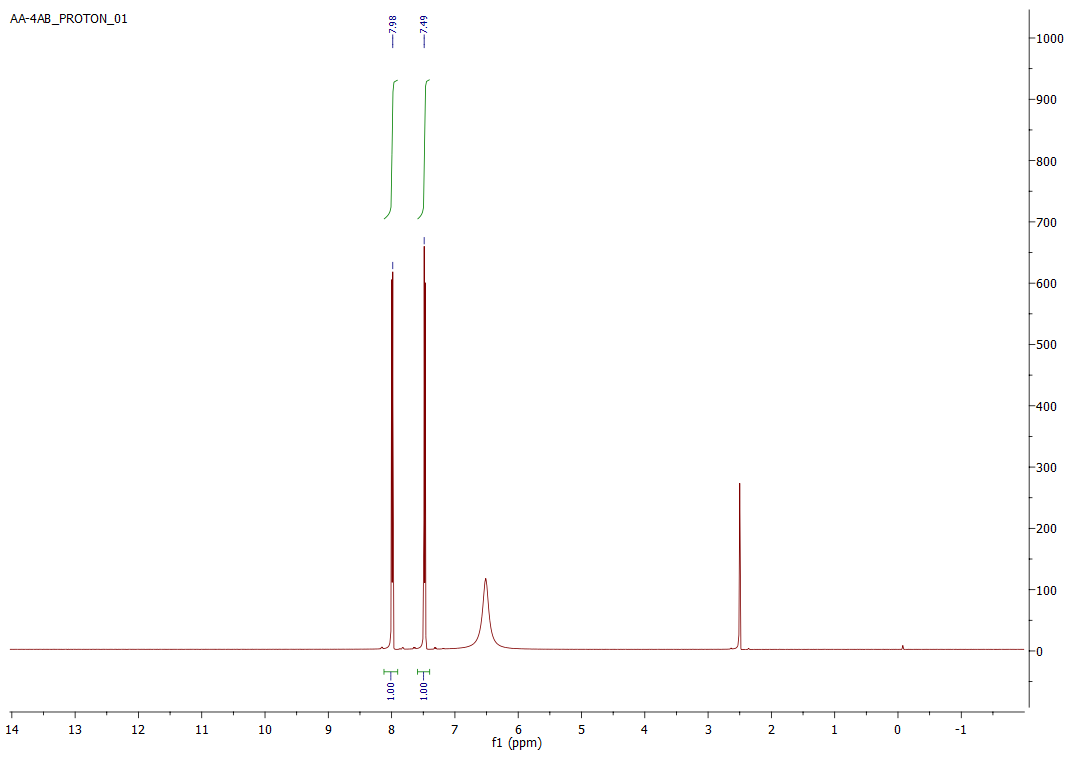


**Figure S8.** ^1^H-NMR of 4-aminobenzoic acid under digestion conditions.


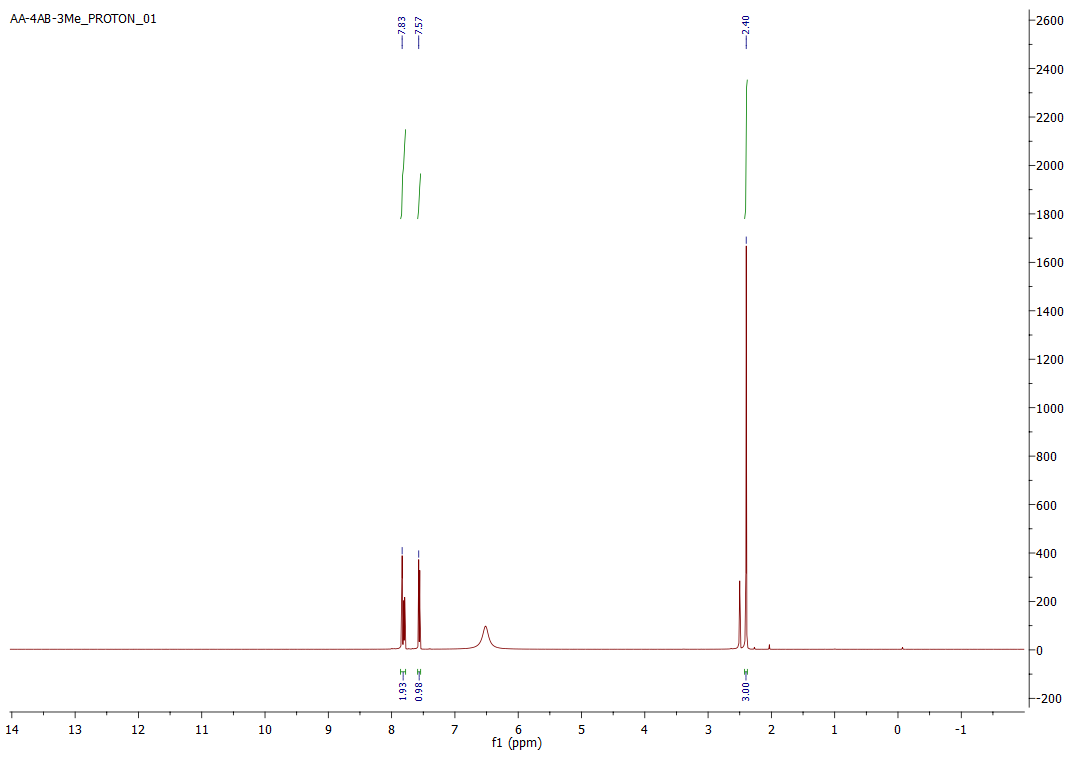


**Figure S9.** ^1^H-NMR of 4-amino-3-methylbenzoic acid under digestion conditions.


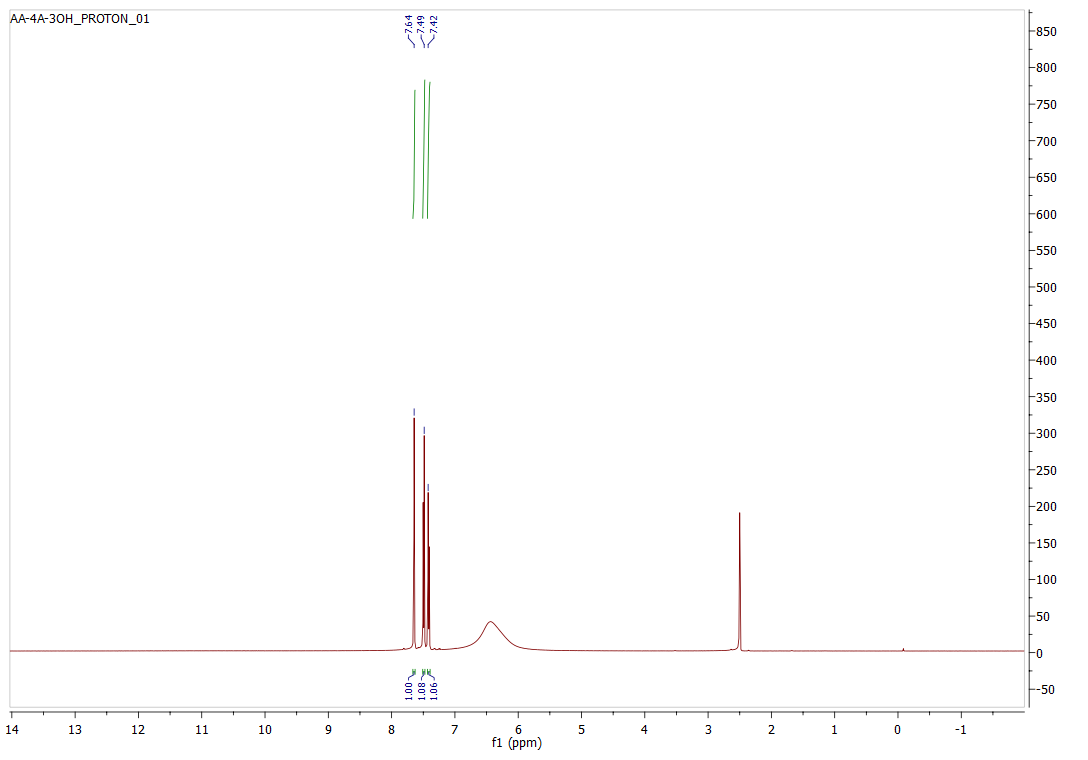


**Figure S10.** ^1^H-NMR of 4-amino-3-hydroxybenzoic acid under digestion conditions.


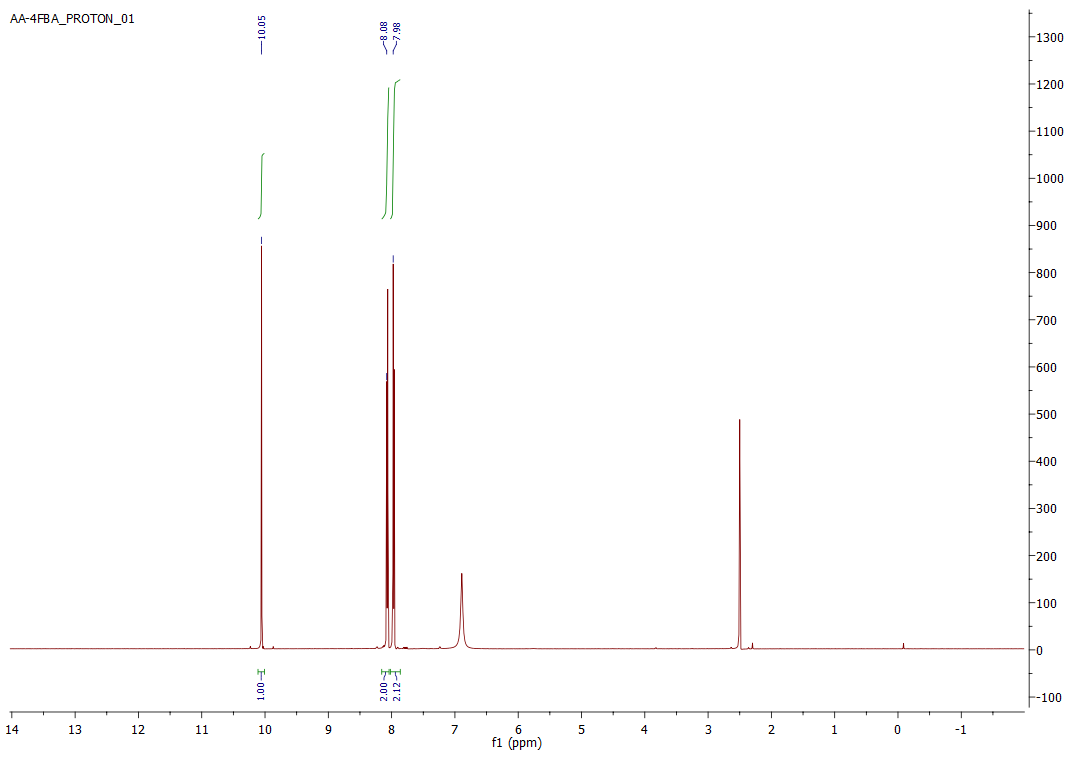


**Figure S11.** ^1^H-NMR of 4-formylbenzoic acid under digestion conditions.


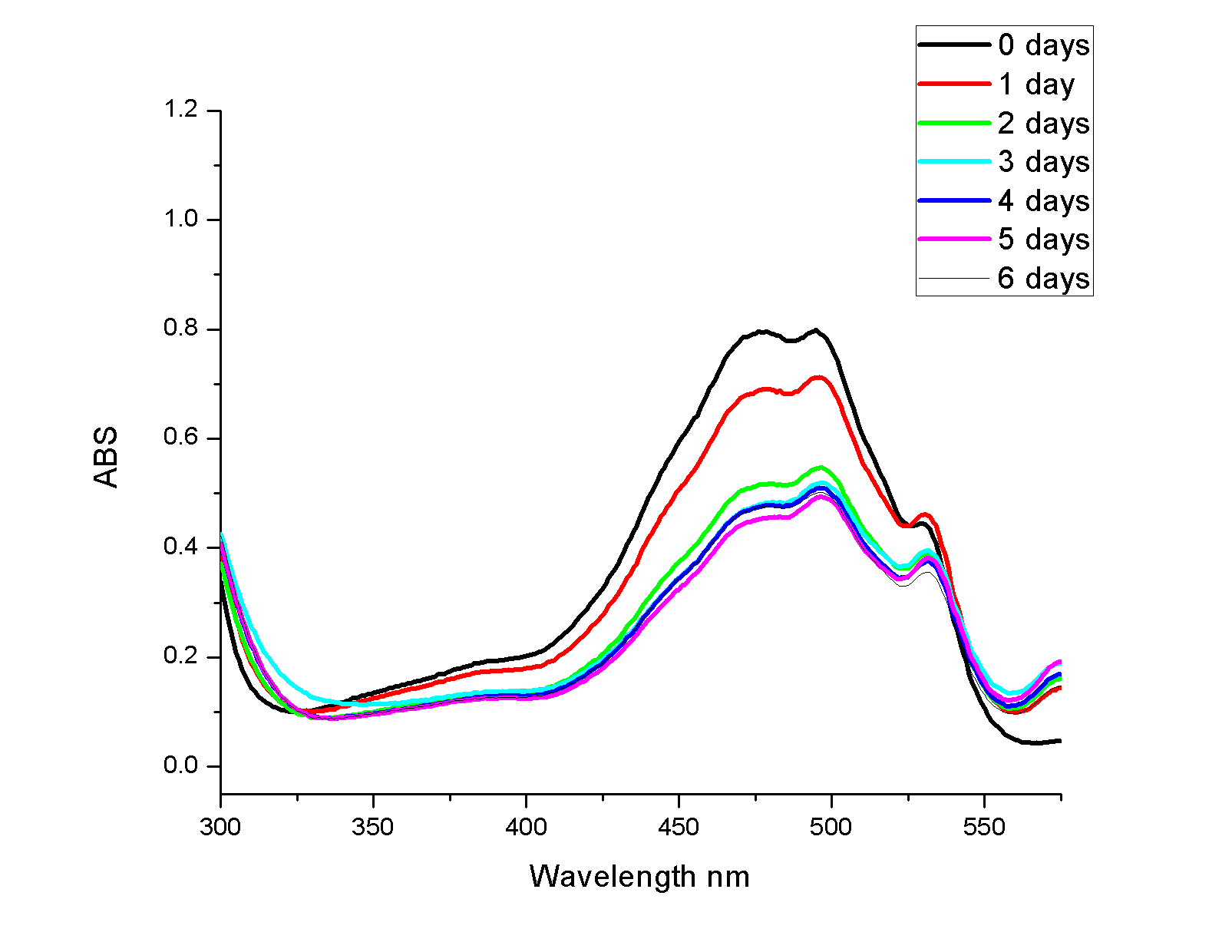


**Figure S12.** UV-Vis data for Dox uptake by **MV-NUIG4-H_50_-OH_50_**.


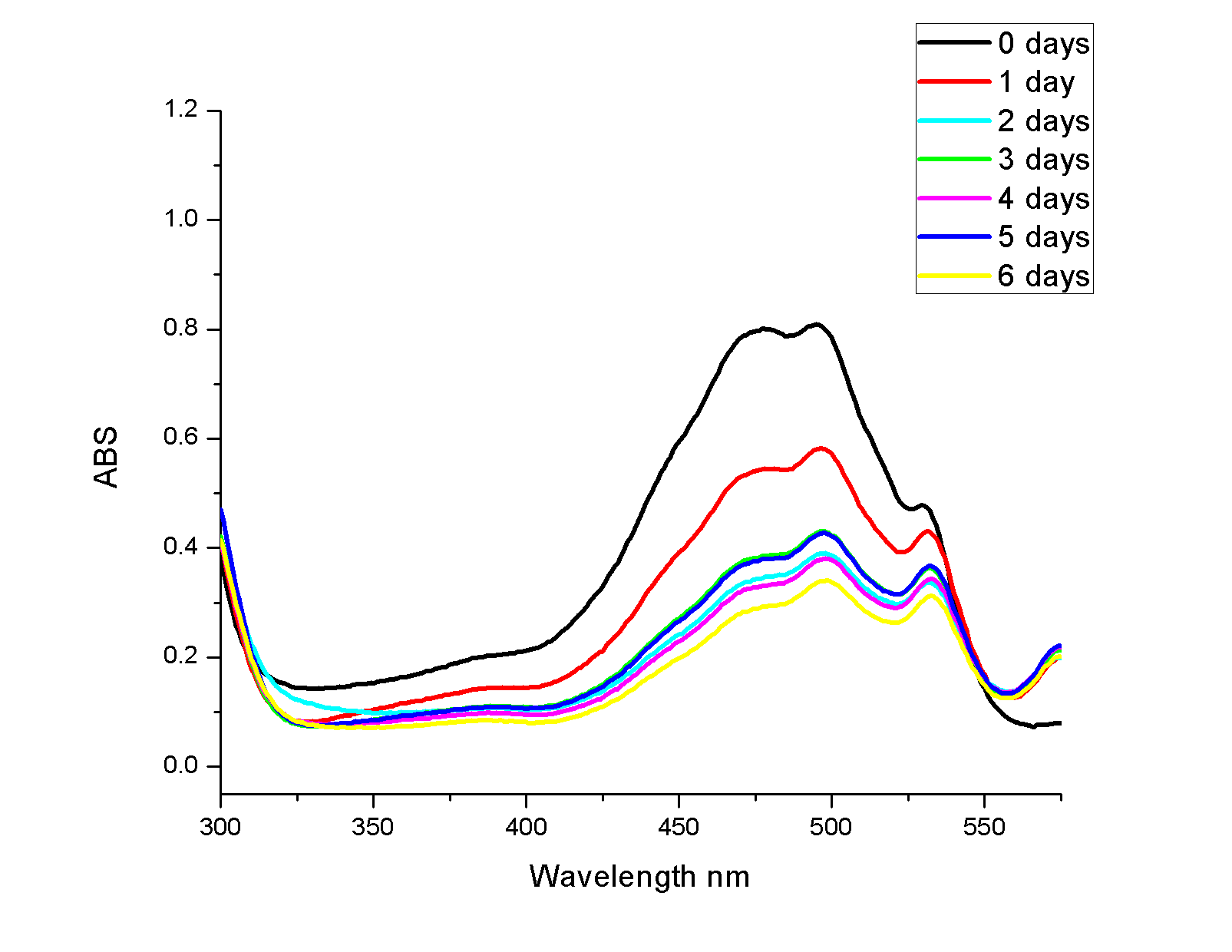


**Figure S13.** UV-Vis data for Dox uptake by **MV-NUIG4-H_90_-OH_10_**.


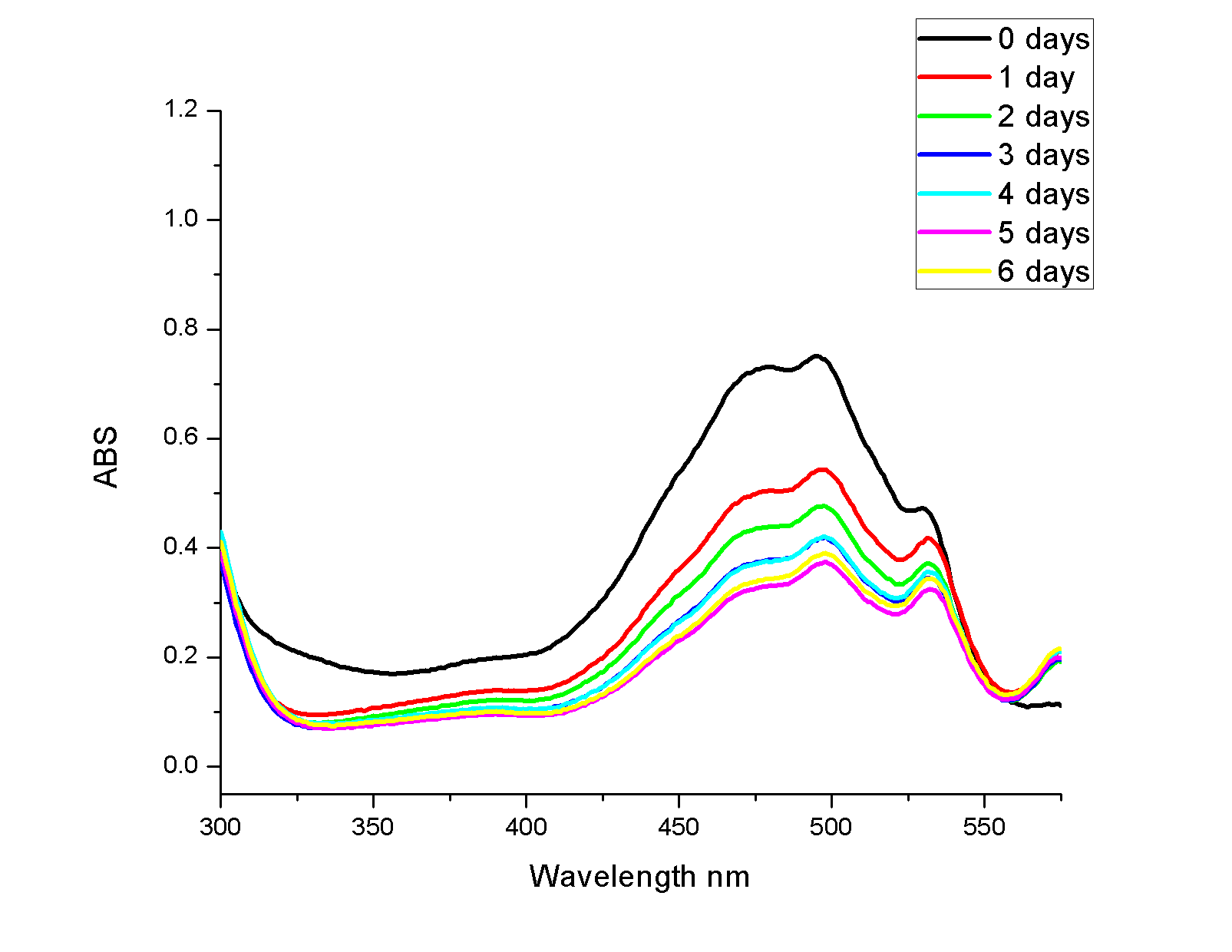


**Figure S14.** UV-Vis data for Dox uptake by **MV-NUIG4-H_75_-OH_25_**.


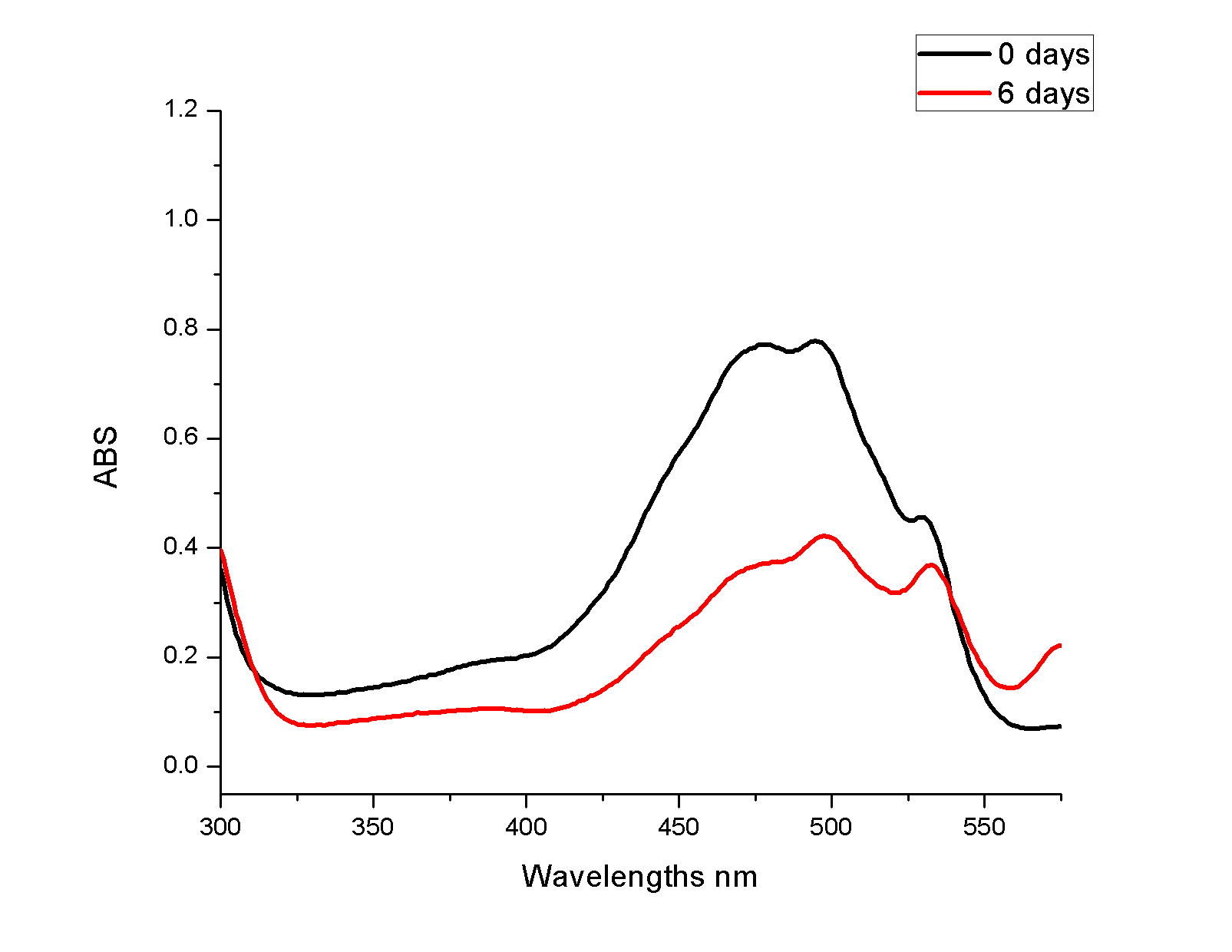


**Figure S15.** UV-Vis data for Dox uptake by **NUIG4-Me**.


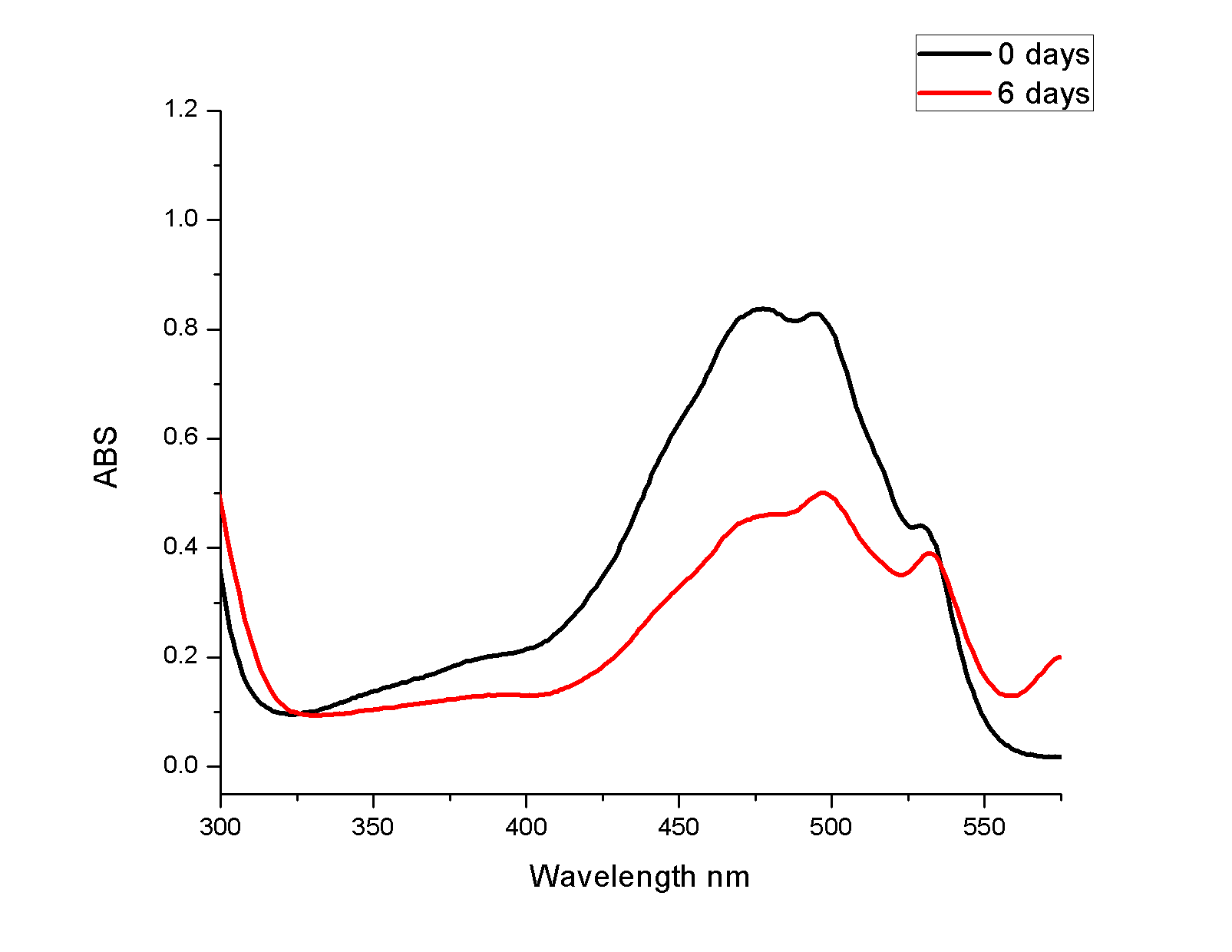


**Figure S16.** UV-Vis data for Dox uptake by **MV-NUIG4-H_50_-Me_50_**.


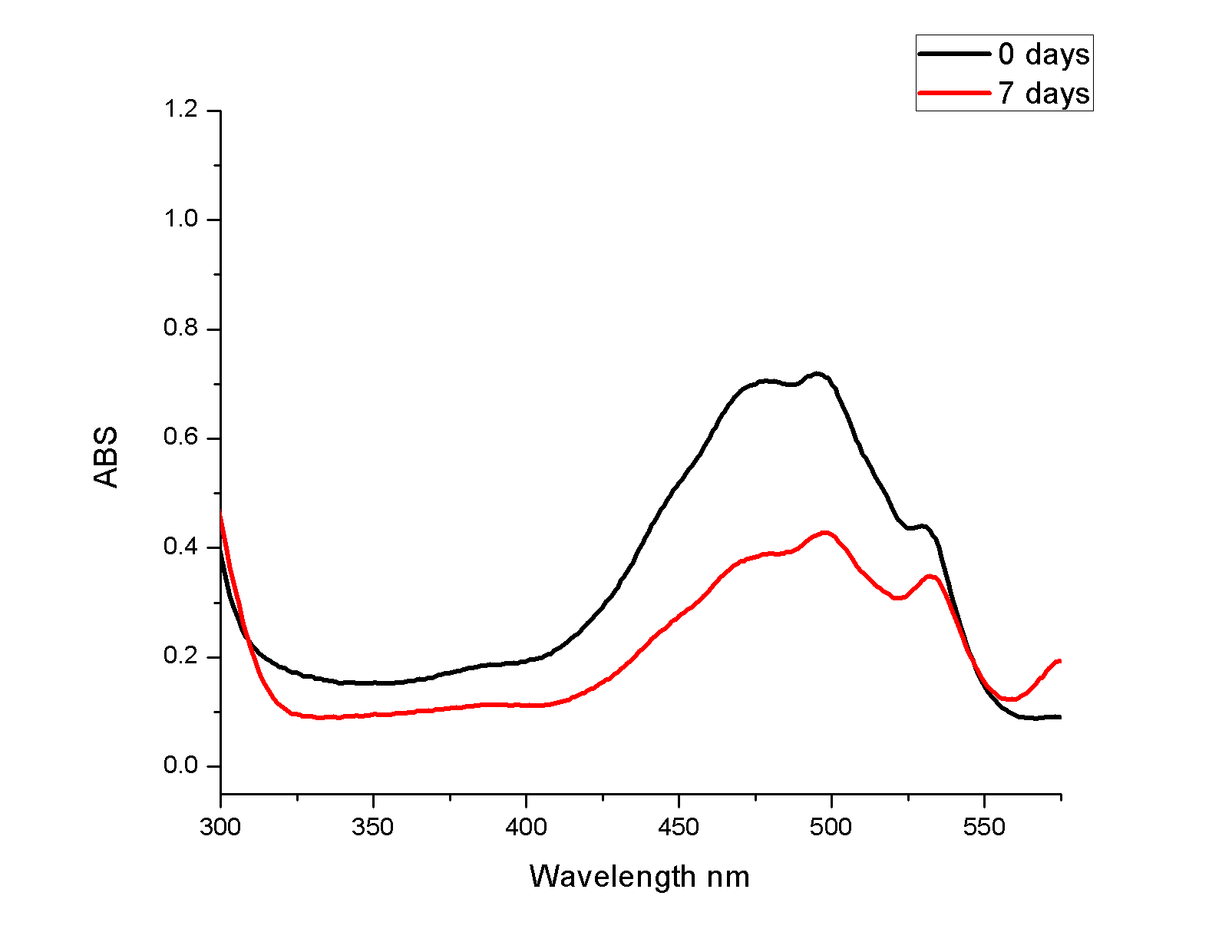


**Figure S17.** UV-Vis data for Dox uptake by **MV-NUIG4-H_90_-Me_10_**.


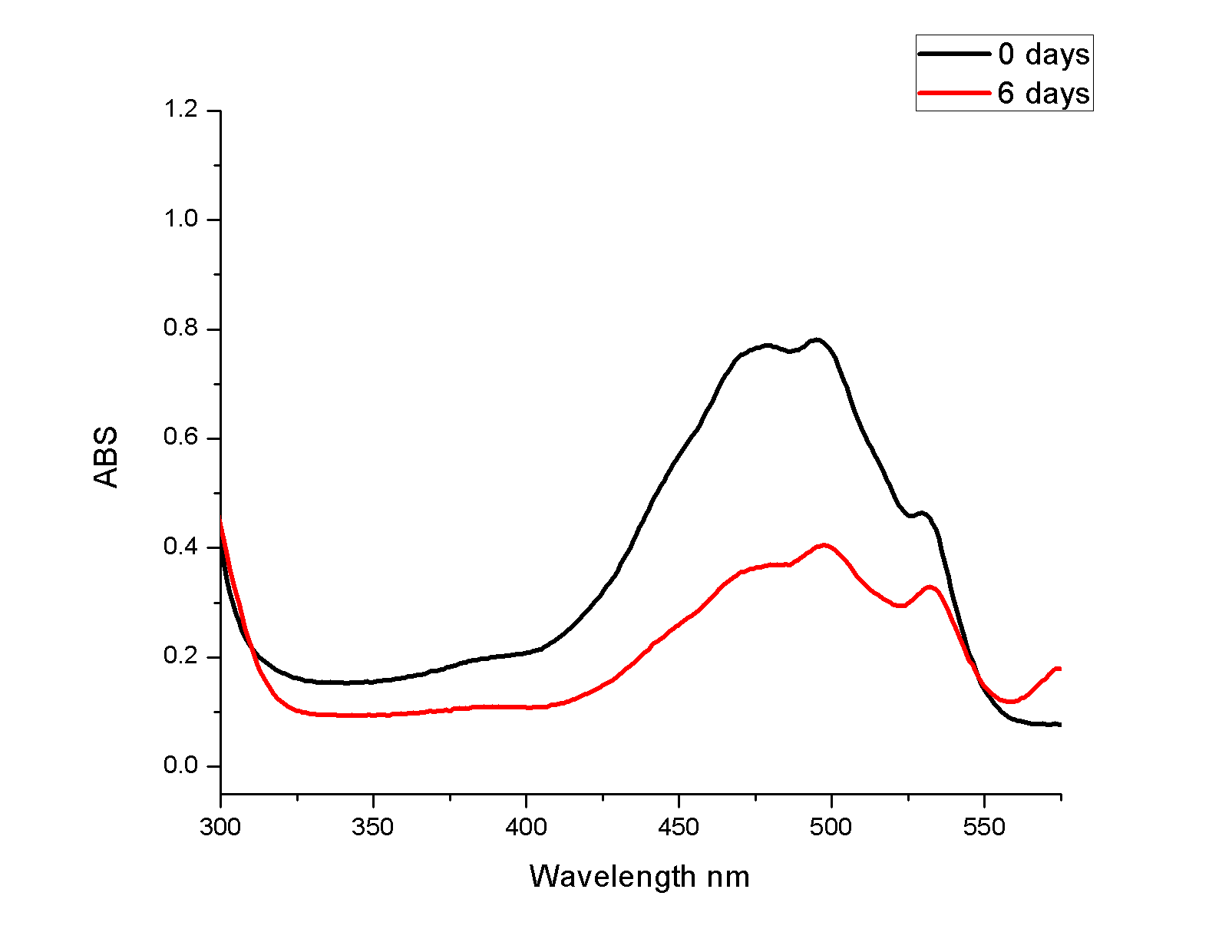


**Figure S18.** UV-Vis data for Dox uptake by **MV-NUIG4-H_75_-Me_25_**.


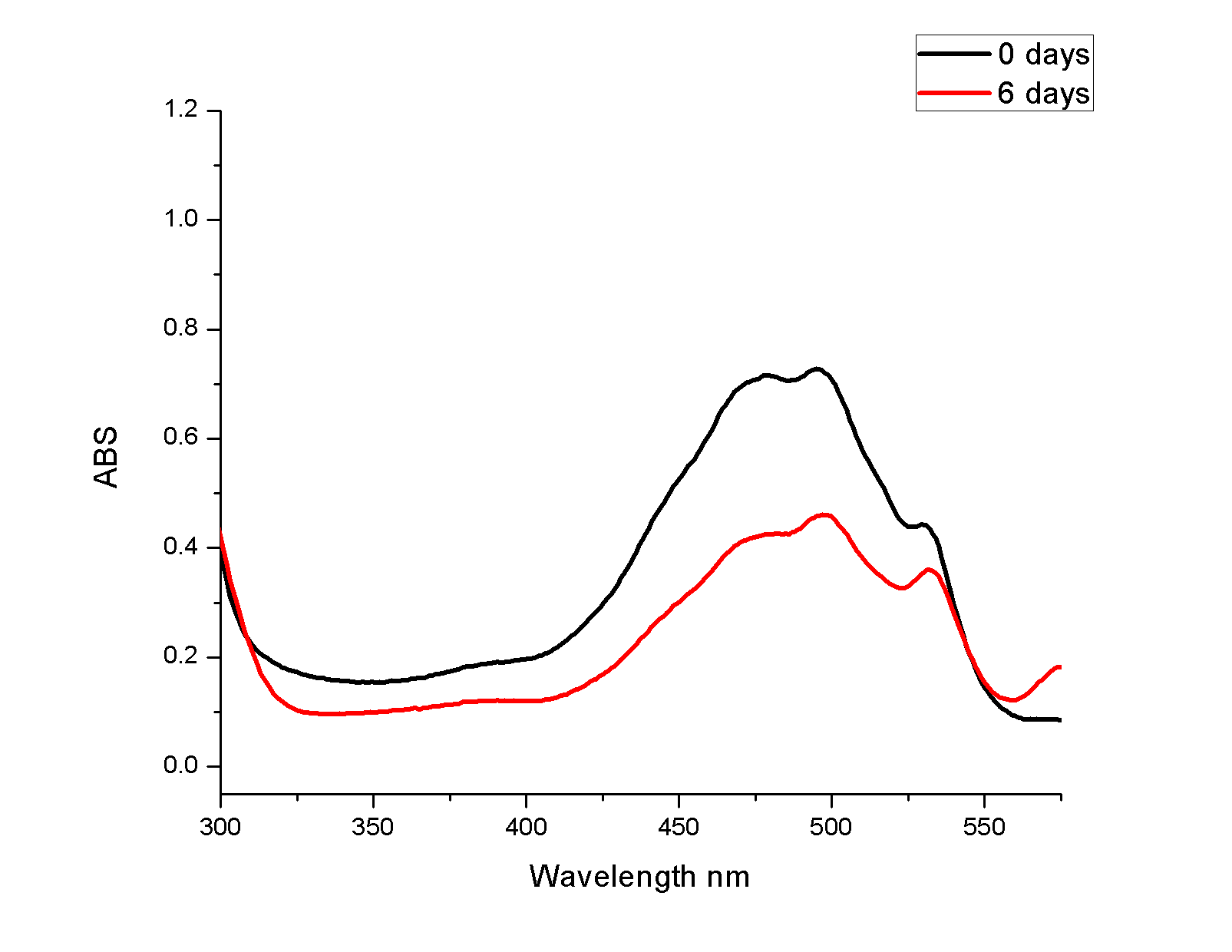


**Figure S19.** UV-Vis data for Dox uptake by **MV-NUIG4-H_25_-Me_75_**.


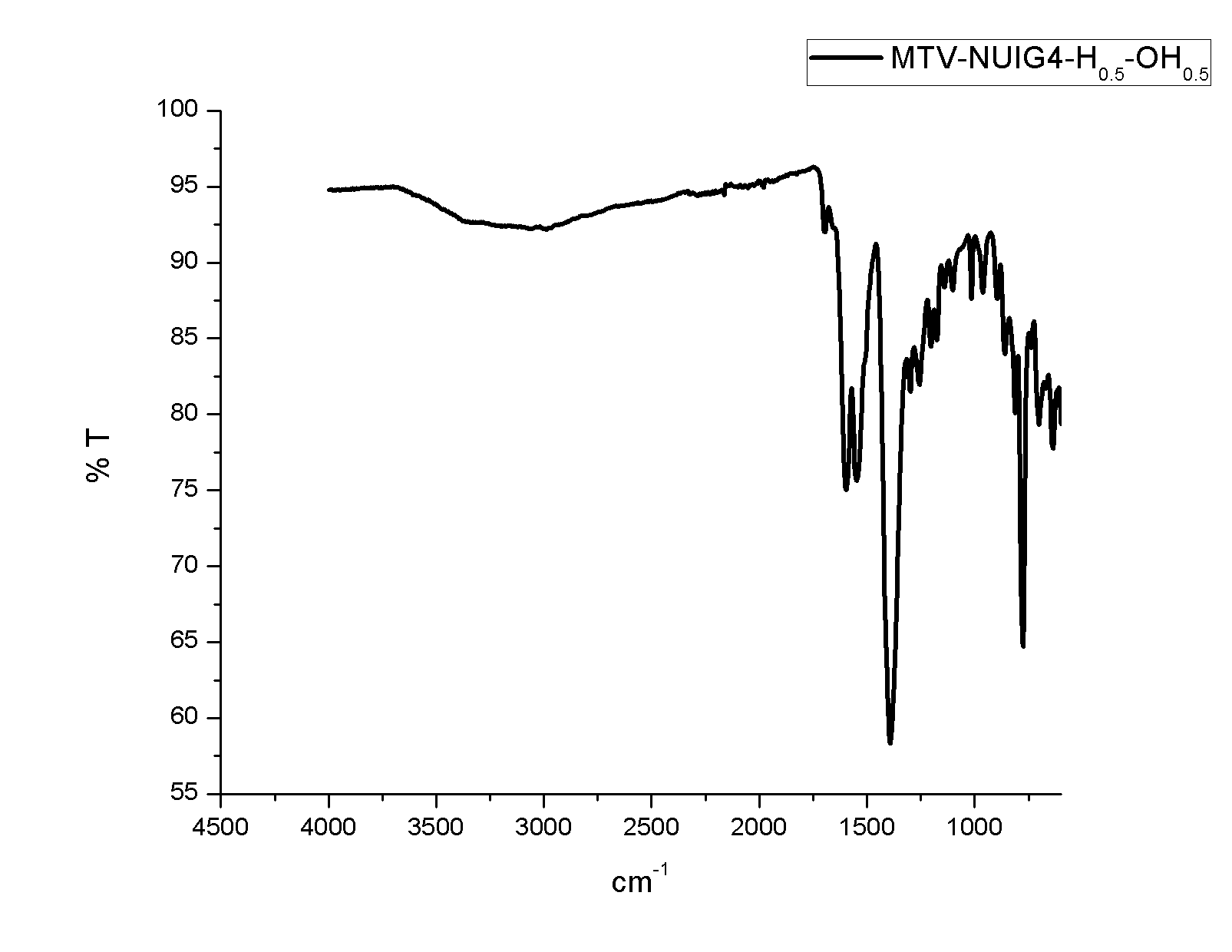


**Figure S20.** FTIR spectrum of **MV-NUIG4-H_50_-OH_50_**.


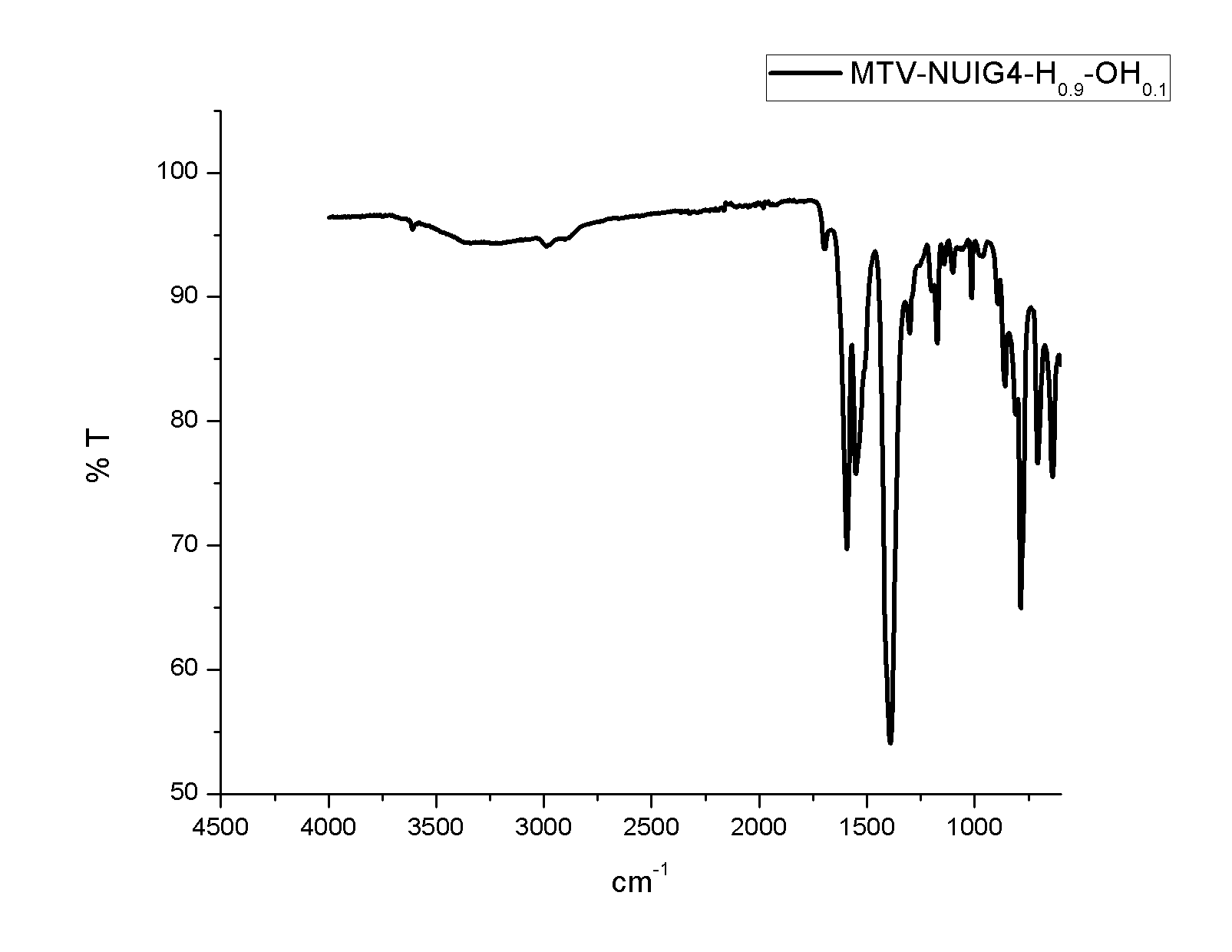


**Figure S21.** FTIR spectrum of **MV-NUIG4-H_90_-OH_10_**.


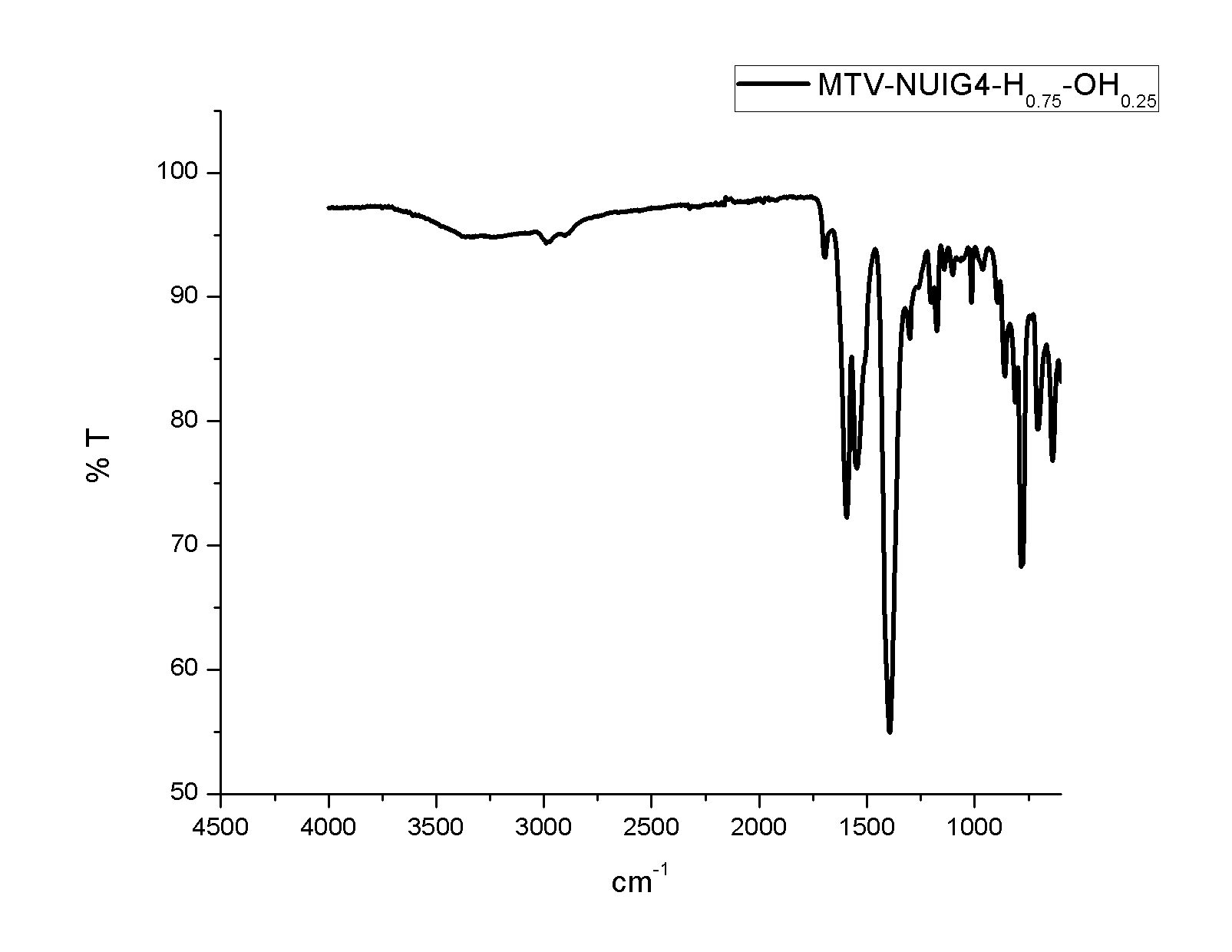


**Figure S22.** FTIR spectrum of **MV-NUIG4-H_75_-OH_25_**.


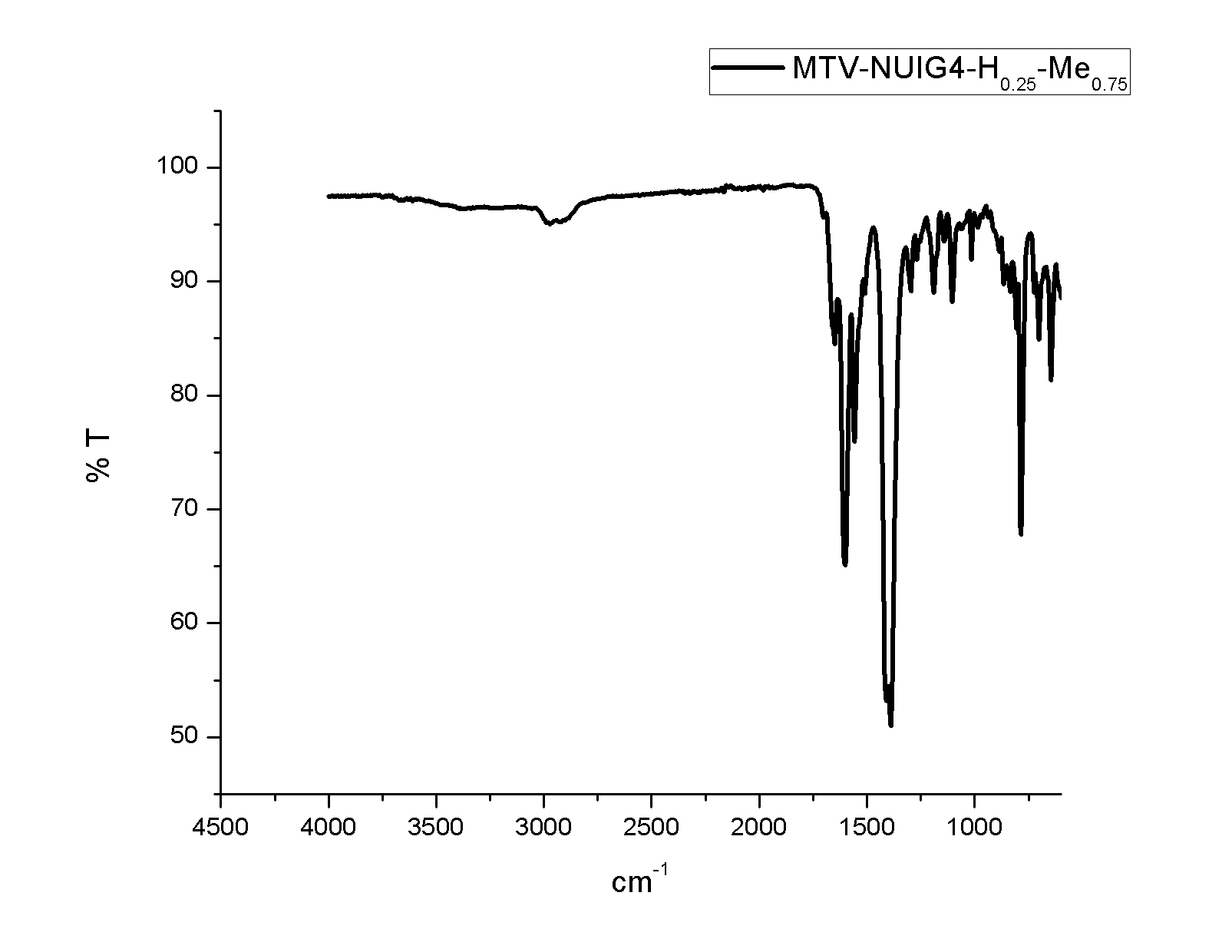


**Figure S23.** FTIR spectrum of **MV-NUIG4-H_25_-OH_75_**.


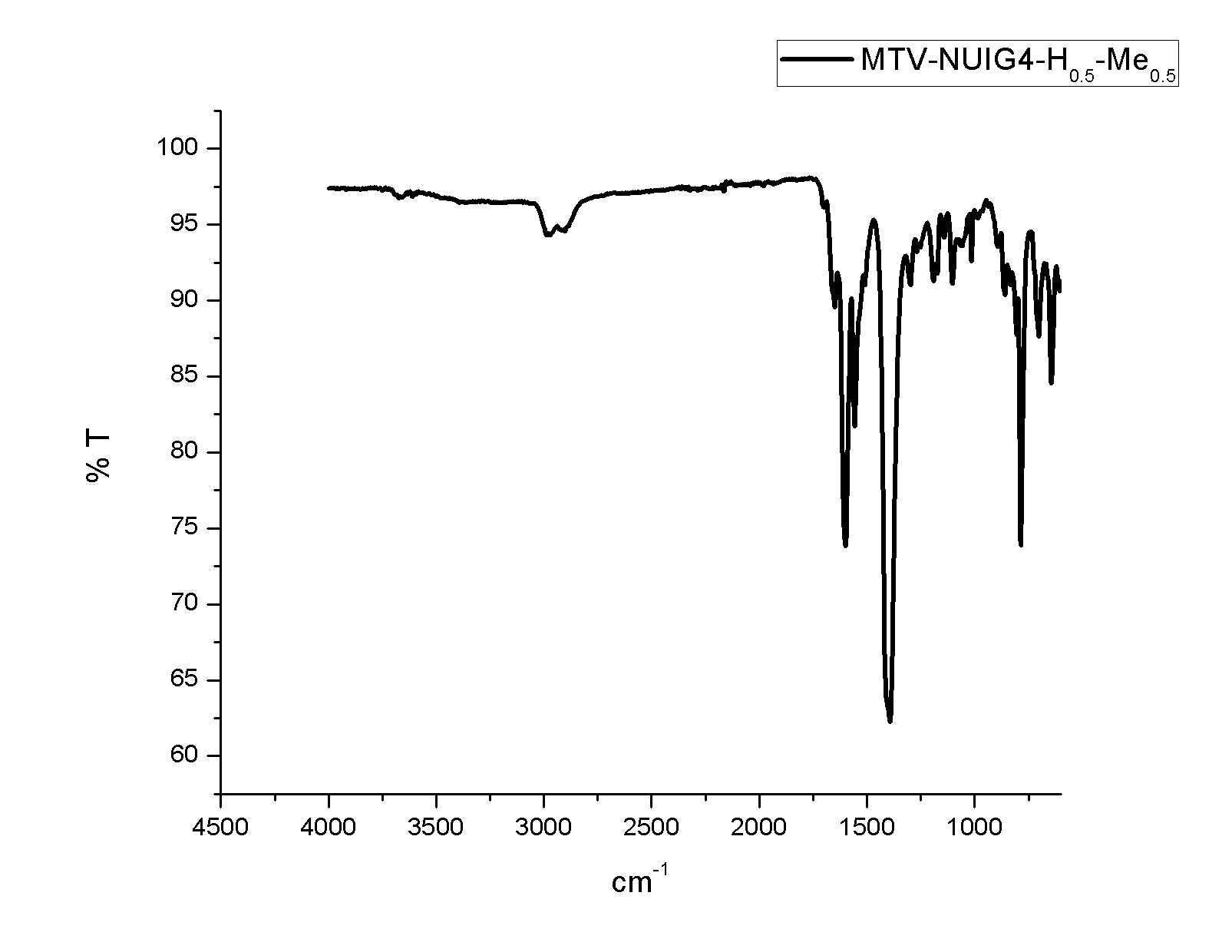


**Figure S24.** FTIR spectrum of **MV-NUIG4-H_50_-Me_50_**.


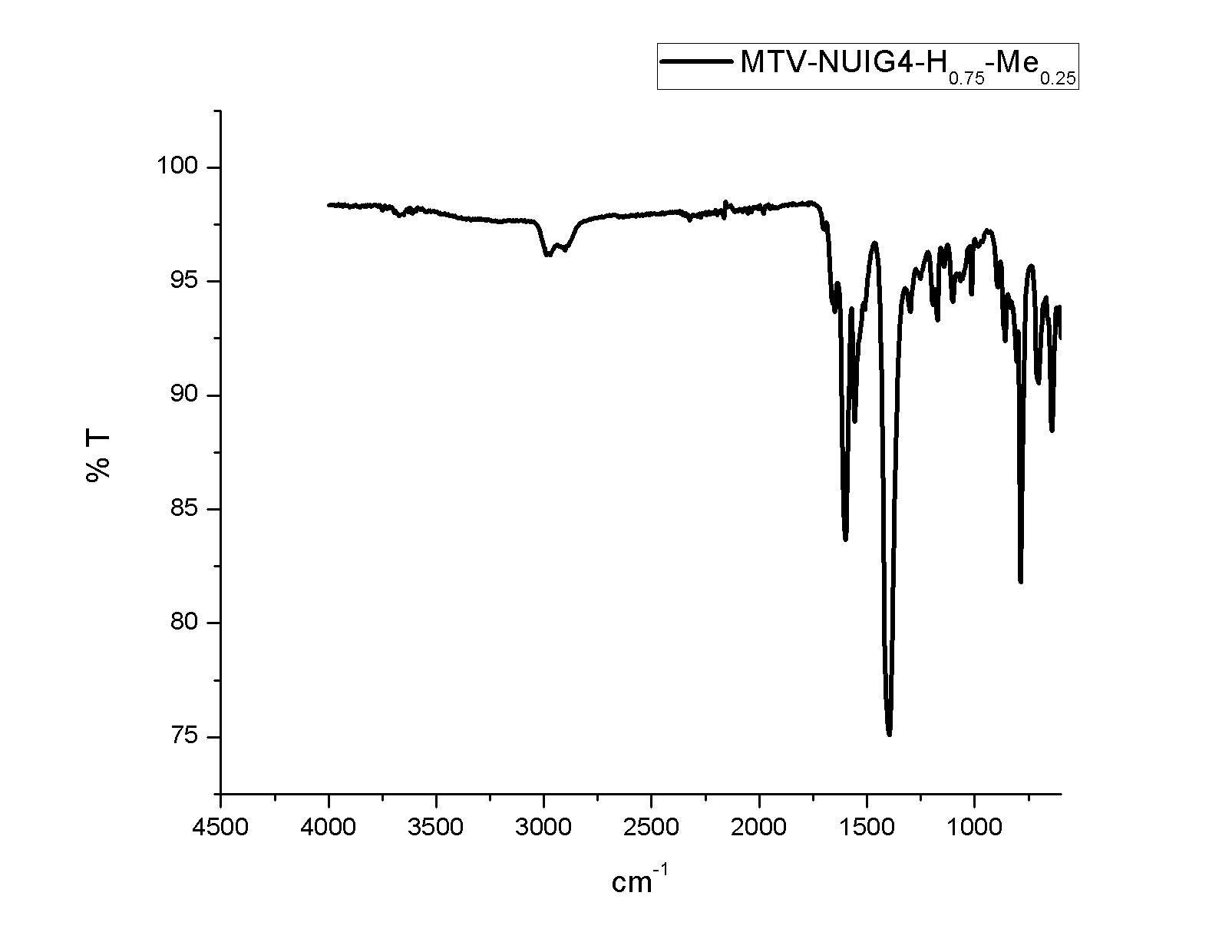


**Figure S25.** FTIR spectrum of **MV-NUIG4-H_75_-Me_25_**.


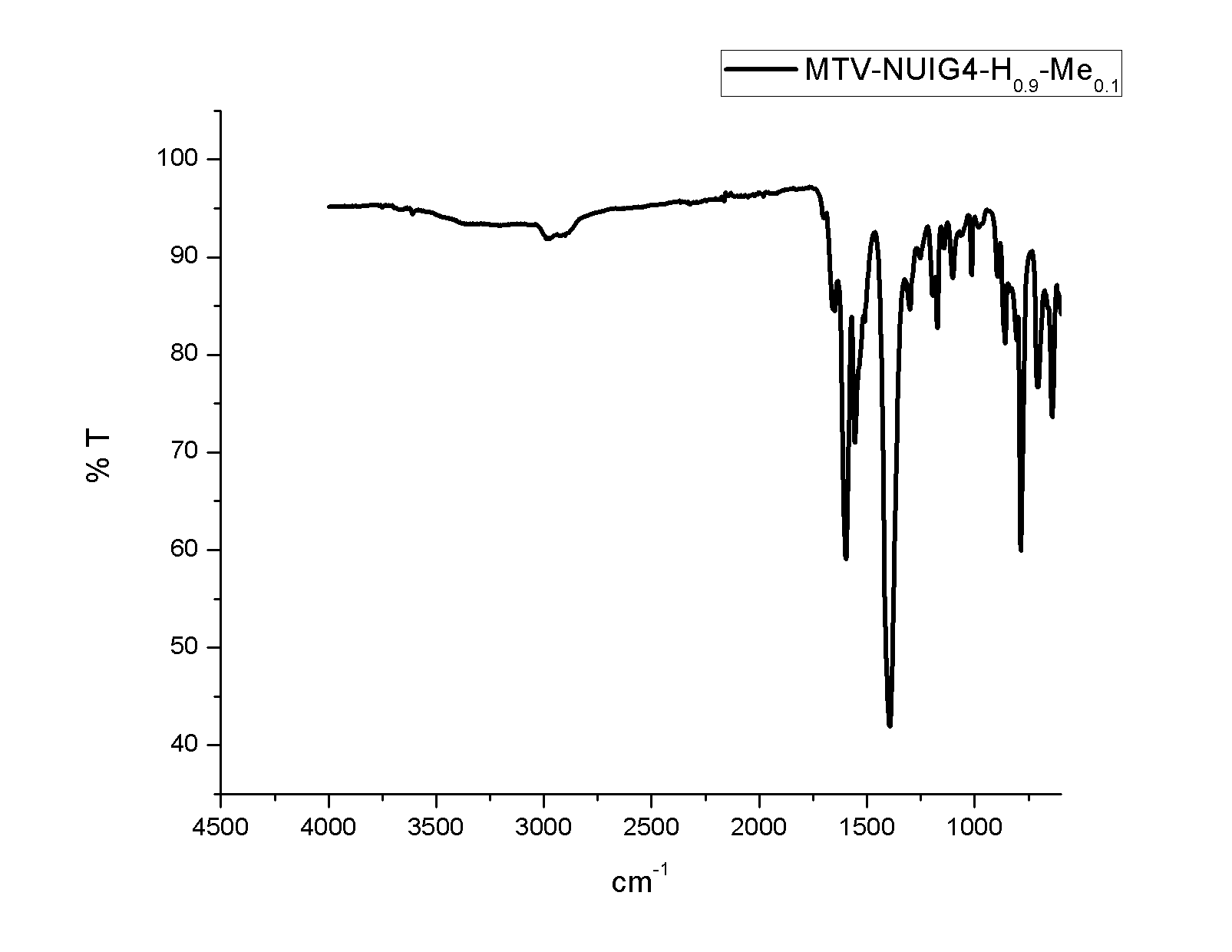


**Figure S26.** FTIR spectrum of **MV-NUIG4-H_90_-Me_10_**.


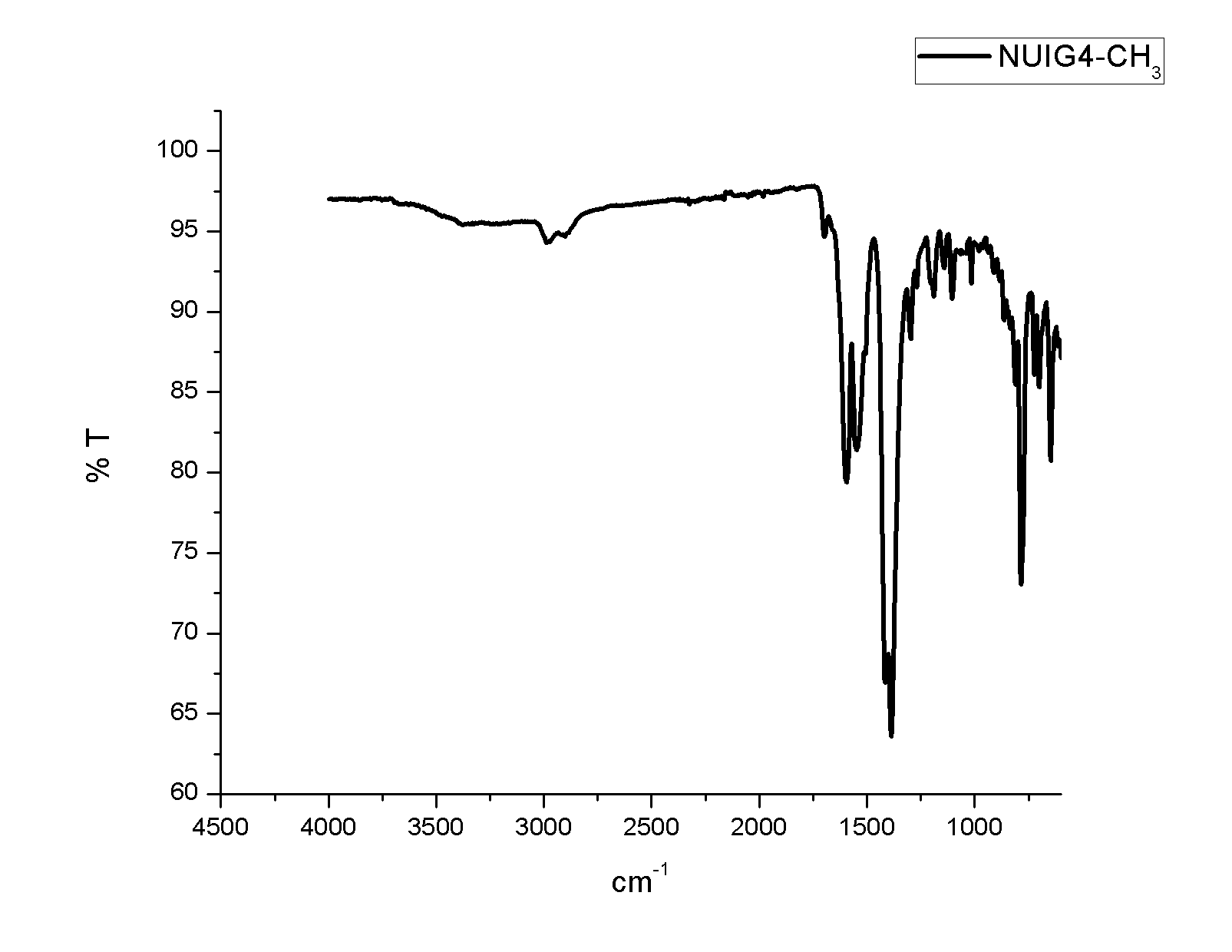


**Figure S27.** FTIR spectrum of **NUIG4-Me**.


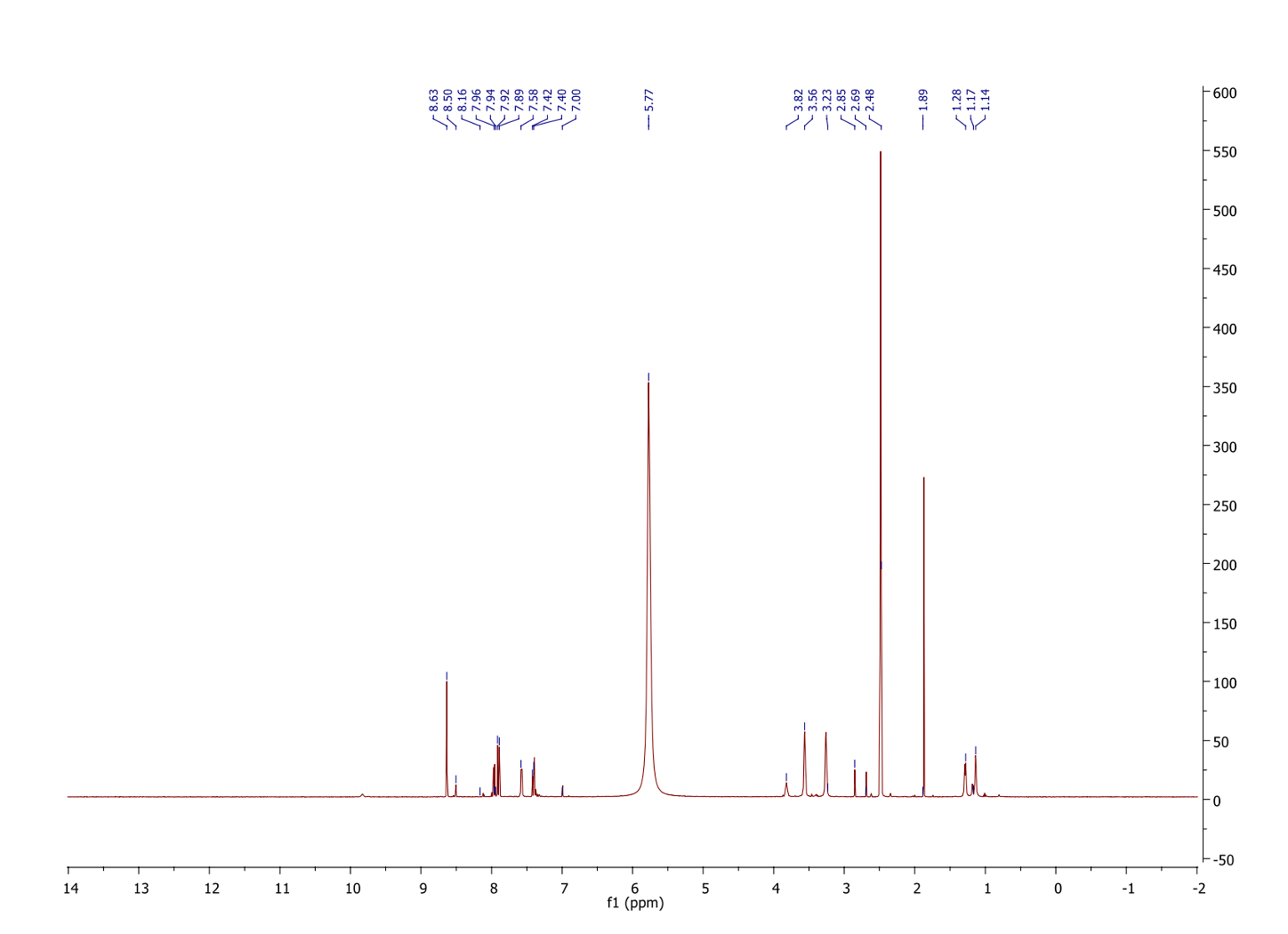


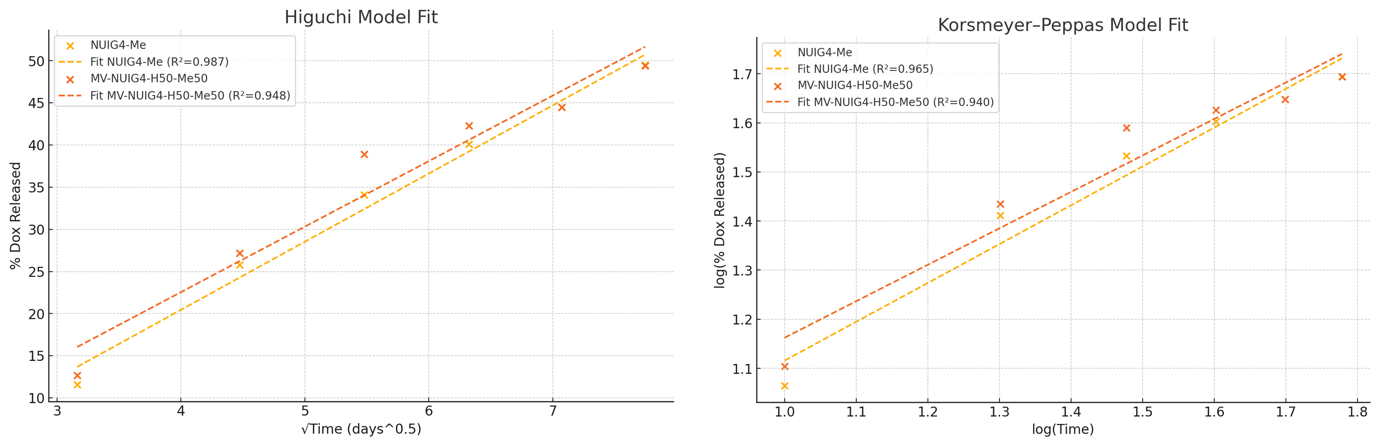
 **Figure S28**. Higuchi (left) and Korsmeyer–Peppas model fitting (right) of Doxorubicin release from **NUIG4-Me** and **MV-NUIG4-H_50_-Me_50_** (pH 5.5).
